# CD36 inhibition reduces non-small-cell lung cancer development through AKT-mTOR pathway

**DOI:** 10.1007/s10565-024-09848-7

**Published:** 2024-02-06

**Authors:** Hui Liu, Wentong Guo, Tianxiang Wang, Peichang Cao, Tingfeng Zou, Ying Peng, Tengteng Yan, Chenzhong Liao, Qingshan Li, Yajun Duan, Jihong Han, Baotong Zhang, Yuanli Chen, Dahai Zhao, Xiaoxiao Yang

**Affiliations:** 1 Department of Respiratory and Critical Care Medicine, The Second Affiliated Hospital of Anhui Medical University, Hefei, China; 2 Key Laboratory of Metabolism and Regulation for Major Diseases of Anhui Higher Education Institutes, Anhui Provincial International Science and Technology Cooperation Base for Major Metabolic Diseases and Nutritional Interventions, College of Food and Biological Engineering, Hefei University of Technology, Hefei, China; 3 College of Life Sciences, Key Laboratory of Medicinal Chemical Biology, Key Laboratory of Bioactive Materials of Ministry of Education, Nankai University, Tianjin, China; 4 Department of Human Cell Biology and Genetics, School of Medicine, Southern University of Science and Technology, Shenzhen, 518055 China

**Keywords:** Lung cancer, CD36, Pitavastatin, FFA, AKT/mTOR pathway

## Abstract

Lung cancer is the most common cause of cancer-related deaths worldwide and is caused by multiple factors, including high-fat diet (HFD). CD36, a fatty acid receptor, is closely associated with metabolism-related diseases, including cardiovascular disease and cancer. However, the role of CD36 in HFD-accelerated non-small-cell lung cancer (NSCLC) is unclear. *In vivo*, we fed C57BL/6J wild-type (WT) and CD36 knockout (CD36^−/−^) mice normal chow or HFD in the presence or absence of pitavastatin 2 weeks before subcutaneous injection of LLC1 cells. *In vitro*, A549 and NCI-H520 cells were treated with free fatty acids (FFAs) to mimic HFD situation for exploration the underlying mechanisms. We found that HFD promoted LLC1 tumor growth *in vivo* and that FFAs increased cell proliferation and migration in A549 and NCI-H520 cells. The enhanced cell or tumor growth was inhibited by the lipid-lowering agent pitavastatin, which reduced lipid accumulation. More importantly, we found that plasma soluble CD36 (sCD36) levels were higher in NSCLC patients than those in healthy ones. Compared to that in WT mice, the proliferation of LLC1 cells in CD36^−/−^ mice was largely suppressed, which was further repressed by pitavastatin in HFD group. At the molecular level, we found that CD36 inhibition, either with pitavastatin or plasmid, reduced proliferation- and migration-related protein expression through the AKT/mTOR pathway. Taken together, we demonstrate that inhibition of CD36 expression by pitavastatin or other inhibitors may be a viable strategy for NSCLC treatment.

**Graphical abstract:**

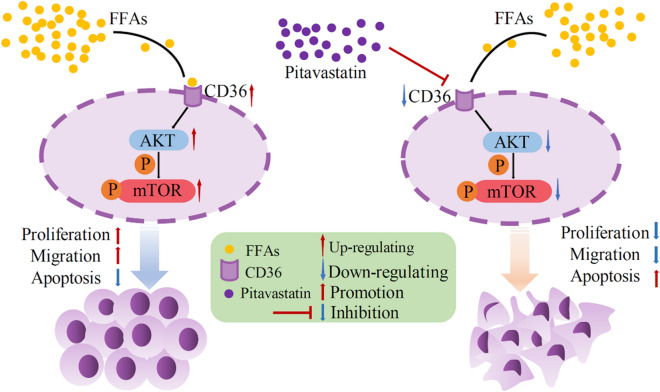

1) Pitavastatin reduces NSCLC progression by inhibiting CD36.

2) Inhibition of CD36 can improve HFD- or FFA-induced NSCLC.

3) AKT/mTOR pathway is involved in CD36-regulated NSCLC.

4) Inhibition of CD36 by pitavastatin or other inhibitors may be a strategy for NSCLC treatment.

**Supplementary information:**

The online version contains supplementary material available at 10.1007/s10565-024-09848-7.

## Introduction

Lung cancer is one of most common cancers and the leading cause of cancer-related death, posing a serious threat to human life. According to the 2020 Global Cancer Statistics, approximately 1.8 million people died from lung cancer worldwide (Sung et al. 2021). The two main subtypes of lung cancer are non-small-cell lung cancer (NSCLC) and small-cell lung cancer. NSCLC accounts for approximately 80 to 85% of lung cancer cases (Yuan et al. 2019). Over the past decade, the therapeutic options for NSCLC have improved. Gefitinib, erlotinib (epidermal growth factor receptor-tyrosine kinase inhibitors, EGFR-TKIs), or crizotinib (anaplastic lymphoma kinase-TKI, ALK-TKI) was chosen for patients with gene mutations in EGFR or ALK. In recent years, immune therapies, such as programmed cell death protein 1 or programmed death ligand 1 inhibitors, have improved outcomes for NSCLC. However, only 1.3–15.4% of patients suffer from NSCLC with *EGFR* and *ALK* mutations, which limits the clinical use of these treatments. The price of immune therapies is too high to afford (Zhuang et al. 2019). Therefore, discovering new targets or therapeutic strategies will greatly benefit lung cancer patients.

Numerous epidemiological studies have demonstrated that an excess of lipids is a risk factor in many types of cancers (Cha and Lee 2016). Lipid metabolism is activated at different stages of cancer. Enhanced lipid uptake and storage can supply energy and substances for tumor cell proliferation, invasion, and metastasis (Bian et al. 2021). In recent years, metabolic regulator factors have been proposed to be therapeutic targets for anticancer drugs. CD36, a receptor for fatty acids, is widely expressed in a variety of cells and participates in multiple signal transduction pathways (Wang and Li 2019). Excess uptake of ox-LDL by CD36 promotes macrophage foam cell formation and initiates lesion development in atherosclerosis (Park 2014). CD36 plays a dominant role in providing sufficient energy to maintain the normal function of cardiac myocytes and pregnant mice by uptake fatty acids (Kim and Dyck 2016; Yang et al. 2016). In addition, an increasing number of studies have shown that upregulated CD36 is associated with tumor progression. In colorectal cancer cells, CD36 enhances the potential for metastasis by regulating matrix metallopeptidase 28 and E-cadherin levels (Drury et al. 2022). In addition to regulating cell proliferation, invasion, and metastasis, CD36 also plays fundamental role in immune system. In tumor-infiltrating CD8^+^ T cells, excess fatty acid uptake is mediated by CD36, leading to reduced cytotoxic cytokine production and impaired antitumor ability (Ma et al. 2021). In addition, CD36 levels are increased in liver cancer metastasis-associated macrophages, which promotes lipid deposition and exert immunosuppressive effects (Yang et al. 2022). Moreover, cancer stem cell of glioblastoma can selectively use CD36 to maintain their renewal and capacity for tumor initiation (Hale et al. 2014). Consistently, our previous study demonstrated that CD36 overexpression facilitates proliferation, migration, and renewal in breast cancer cells (Liang et al. 2018). Therefore, CD36 regulates lipid metabolism reprogramming to promote cancer progression and functions in tumor-associated immune cells to suppress antitumor immune activity. Nevertheless, the role of CD36 in NSCLC is still unclear.

Pitavastatin is a lipid-lowering drug that has been widely used in the clinic for hypercholesterolemia treatment. Our group has demonstrated that statins can also treat hypertriglyceridemia (Zhang et al. 2018). In recent years, pitavastatin has been proven to have an inhibitory role in cancers. In an oral cancer cell line, it increased cell apoptosis in a forkhead box O3A–dependent manner (Tilija Pun et al. 2022). Pitavastatin can suppress cell proliferation and metastasis-linked bone loss in a mevalonate pathway (Wang et al. 2019). As a 3-hydroxy-3-methylglutaryl coenzyme A reductase inhibitor, pitavastatin can also inhibit peroxisome proliferator–activated receptor γ (PPARγ), as well as the downstream target CD36 in macrophages and adipocytes (Nicholson et al. 2007). Considering the fact that lipid dysregulation promotes cancer development, we hypothesized that pitavastatin may be a promising drug for NSCLC treatment, especially under high-fat diet (HFD) conditions. In the current study, we determined the effects of pitavastatin on A549 and NCI-H520 cell viability under normal and HFD condition. Furthermore, cells were transfected with overexpression or knockdown plasmid of CD36 for exploration whether CD36 is involved in pitavastatin-reduced cell proliferation. *In vivo*, we treated wild-type (WT) mice with a normal chow (NC) or HFD for the determination of excess lipids in NSCLC and investigation the role of pitavastatin in reducing cancer. In addition, CD36 knockout (CD36^−/−^) mice were used to verify the role of CD36 in the pitavastatin-mediated suppression of NSCLC.

## Materials and methods

### Reagents

Pitavastatin was purchased from Macklin Biochemical Co., Ltd. (Shanghai, China). LY294002 was purchased from MedChemExpress (New Jersey, USA). MTT, PA, and OA were purchased from Sigma-Aldrich (St. Louis, State of Missouri, USA). Antibodies used in this study are shown in Table S1. Human CD36 ELISA kit was purchased from Ruixin Biotechnology Co., Ltd. (Quanzhou, China). The assay kits of triglyceride (TG), free fatty acid (FFA), and cholesterol were purchased from Wako Pure Chemical Industries, Ltd. (Osaka, Japan), Solarbio Life Sciences (Beijing, China), and Applygen Technologies Inc., respectively.

### Human sample collection

All experiments with human blood and tissue samples were approved by the Ethics Committee of the Second Affiliated Hospital of Anhui Medical University (SL-YX2021-064) and strictly adhered to the Declaration of Helsinki Principle 2008. All people submitted informed consent before sample collection. In this study, we collected blood samples from NSCLC patients (*n* = 14) and healthy people (*n* = 24). And NSCLC tumor tissues (*n* = 5) and adjacent non-cancerous precancerous tissues (*n* = 2) were collected.

### Animals

The protocol for animal study was approved by the Ethics Committee of Hefei University of Technology (HFUT20220101002) and conformed to the Guide for the Care and Use of Laboratory Animals published by NIH. All mice were housed in a chamber with constant humidity of 55 ± 2% and a temperature of 22 ± 2 °C for a cycle of 12-h light and 12-h dark. About 6-week-old male mice were obtained from GemPharmatech Co., Ltd. (Nanjing, China). CD36^−/−^ mice were generated by the GemPharmatech Co., Ltd. using the CRISPR/Cas9 technology to knockout the exon4 of *CD36* transcript. The offspring mice were genotyped by PCR with the following sequences: Cd36-5wt-F: 5′-TTTCCCTAAGACTCTGCTACTATTT-3′; Cd36-5wt-R: 5′-ATGCAAAATCATTTTAGCTCTGTG-3′; Cd36-wt-F: 5′-TCCAGCAATCCTCAAACATA-3′; and Cd36-wt-R: CCTTTGGCAACACTCCCTTA.

In the current study, mice were fed with normal diet (10% kcal from fat) or HFD (42% kcal from fat) and intraperitoneally (i.p.) injected with pitavastatin calcium (2 mg/kg in normal saline/NS) or NS for 5 weeks. At the third week, LLC1 cells (1 × 10^6^) were inoculated subcutaneously into the left side of the mouse’s rib cage and tumor growth was measured every 3 days. Volume of the xenograft tumor was calculated by the following standard formula: length × width × width × 0.5 (Wang et al. 2016). At the end of the experiment, all mice were euthanized, and blood and tumor and lung tissues were collected for further analysis.

### Cell culture

A549 and LLC1 cells were purchased from ATCC (Manassas, VA, USA). NCI-H520 cells were kindly provided by Dr. P. Luo. A549 and NCI-H520 cells were cultured in Dulbecco’s modified Eagle’s medium (DMEM, Biological Industries, Kibbutz Beit Haemek, Israel), and LLC1 cells were cultured in DMEM without sodium pyruvate medium containing 10% fetal bovine serum (FBS, AusGeneX, Brisbane, AUS) and 50 µg/mL penicillin and streptomycin (Hyclone, Logan, USA). Cells received indicated treatment at ~ 80% confluence.

### FFA preparation

PA solution was firstly prepared in 50% ethanol solution to make stock solution (150 mM) and then mixed with 5% BSA to obtain 7.5 mM PA working solution. The 15 mM OA working solution was obtained by being diluted with 5% BSA. The mixture of PA and OA was obtained at a volume ratio of 1:1 of working solution, which was named as FFA solution (Del Bo et al. 2016).

### Preparation and transfection of overexpression or knockdown plasmids

The plasmid for CD36 overexpression was constructed in our previous study and named as pCMV-CD36 (Liang et al. 2018). For overexpression experiment, cells were transfected with plasmids of pCMV or pCMV-CD36 overnight using Hieff Trans™ Reagent (Yeasen Biotechnology Co., Ltd., shanghai, China), followed by further treatment.

CD36-knockdown vector was constructed using CRISPR-CasRx methods. Simply, the sequence of gRNA was designed as follows: forward, 5′-AAACACACAGGGATTCCTTTCA GATT-3′ and backward, 5′-AAAAAATCTGAAAGGAATCCCTGTGT-3′. CasRx gRNA cloning backbone (pXR003, #109053, Addgene) was digested with BbsI and then ligated with annealed oligo duplex using T4 ligase. The obtained CasRx-CD36 plasmid was verified with U6 sequencing primer (Konermann et al. 2018). For knockdown experiment, cells at ~ 60% confluence were transfected with EF1a-CasRx-2A-EGFP (pXR001, #109049, Addgene) and CasRx gRNA cloning backbone or CasRx-CD36 plasmid for 12 h using Hieff Trans™ Liposomal Transfection Reagent and then received the indicated treatment.

### Determination of cell viability

Cells in a 96-well plate (7 000 cells/well) were cultured for 12 h and then received the indicated treatment. After treatment, MTT solution (100 µL/well, 0.5 mg/mL) was added to the cells and incubated for 4 h. Then, cells were washed with PBS twice followed by adding DMSO (100 µL). The absorbance was measured at the wavelength of 570 nm.

### Determination of cell cycle and apoptosis by flow cytometry

To conduct the assay of cell cycle, cells in six-well plate were collected after treatment, washed, and resuspended with PBS. Cell suspension was added dropwise to 70% ethanol and fixed at − 20 °C for more than 12 h. Then, cells were washed with PBS and incubated with the solution containing 10 mg/mL RNase and 1 mg/mL propidium iodide for 20 min in the dark. Phases of cell cycle were determined by flow cytometry (Liang et al. 2018).

For apoptosis assay, cells in six-well plates were collected after treatment and stained according to instruction of Annexin V-FITC/PI apoptosis detection assay kit (Yeasen Biotechnology Co., Ltd., Shanghai, China). Composition of apoptotic cells was analyzed by flow cytometry.

### Cell migration was determined using the assay of cell scratch

Cells (~ 80% confluence) in the 12-well plate were damaged by drawing a cross in center of the well with a 200 µL plastic tip. The suspended cells and cell debris was removed by washing with PBS. Cells were cultured and photographed. We recorded the width of scratching and named it as *W*_0_. After treatment for 48 h, we recorded the scratch width again and named it as *W*_48_. The migration rate was calculated as (*W*_0_ − *W*_48_)/*W*_0_ × 100%.

### Determination of cellular or serum lipid profiles

For determination of TG content, cells or tumor tissue was homogenized in PBS. We used a portion of homogenate to determine protein content, and the other to extract total lipids. Briefly, 1 mL lysate was mixed with the 1.5 mL methanol-chloroform (2:1) mixture and vortexed and then added with an additional 0.5 mL chloroform, followed by centrifugation for 5 min at 3000 rpm. Lipid was contained in the organic phase and transferred to a new tube. N_2_ was used to dry the lipids, and 2-propanol was for re-dissolution of lipids. TG content was analyzed using the indicated kit. For determination of FFA and cholesterol content, cell or tumor tissue was homogenized in lysate reagent, and FFA and cholesterol levels were determined according to the manufacturer’s instruction by the indicated assay kits.

For determination serum lipid profiles, blood samples were collected and kept at RT for 2 h and then centrifuged for 20 min at 2000 g. The supernatant was transferred to a new tube and stored at the fridge of − 80 °C. Serum T-CHO, LDL-C, HDL-C, TG, and FFA levels were measured using the biochemical analyzer (Model 7020, Hitachi, Tokyo, Japan).

### H&E staining

Tumor tissue was fixed in 4% paraformaldehyde for 24 h and then dehydrated using an automatic dehydrator (Leica, Wisral, Hesse-Darmstadt, Germany). Tissue samples were embedded in paraffin and used for preparation 5 µm sections. Sections were used for conducting H&E staining to determine metastases (Wang et al. 2016). Images were captured by a fluorescence microscope of ZEISS Scope A1. The tumor area was quantified using the software of ImageJ.

### Protein determination by Western blot and immunohistochemistry (IHC) staining

Cell or tissue total protein was collected, ~ 60 µg protein was used for determining the expression of CD36, PCNA, CDH1, vimentin, Bcl-2, BAX, AKT, mTOR, p-AKT, and p-mTOR by Western blot with indicated antibodies, and the dilution is listed in Table S1 (Wang et al. 2016). Tumor sections were conducted IHC staining for determining CD36, Ki-67, BAX, vimentin, p-AKT, and p-mTOR expressions as described (Yang et al. 2015), and the dilution of antibody is listed in Table S1.

### Statistical analysis

The data were obtained from at least three independent experiments and expressed as mean ± SEM. All data were statistically analyzed by GraphPad Prism 8.0 software, and the statistical analysis was performed by one-way analysis of variance (ANOVA) or *t*-test. Significant difference was considered when *p* < 0.05.

## Results

### Pitavastatin inhibits exogenous free fatty acid (FFA)-enhanced proliferation and migration but promotes apoptosis in vitro

**Fig. 1:**
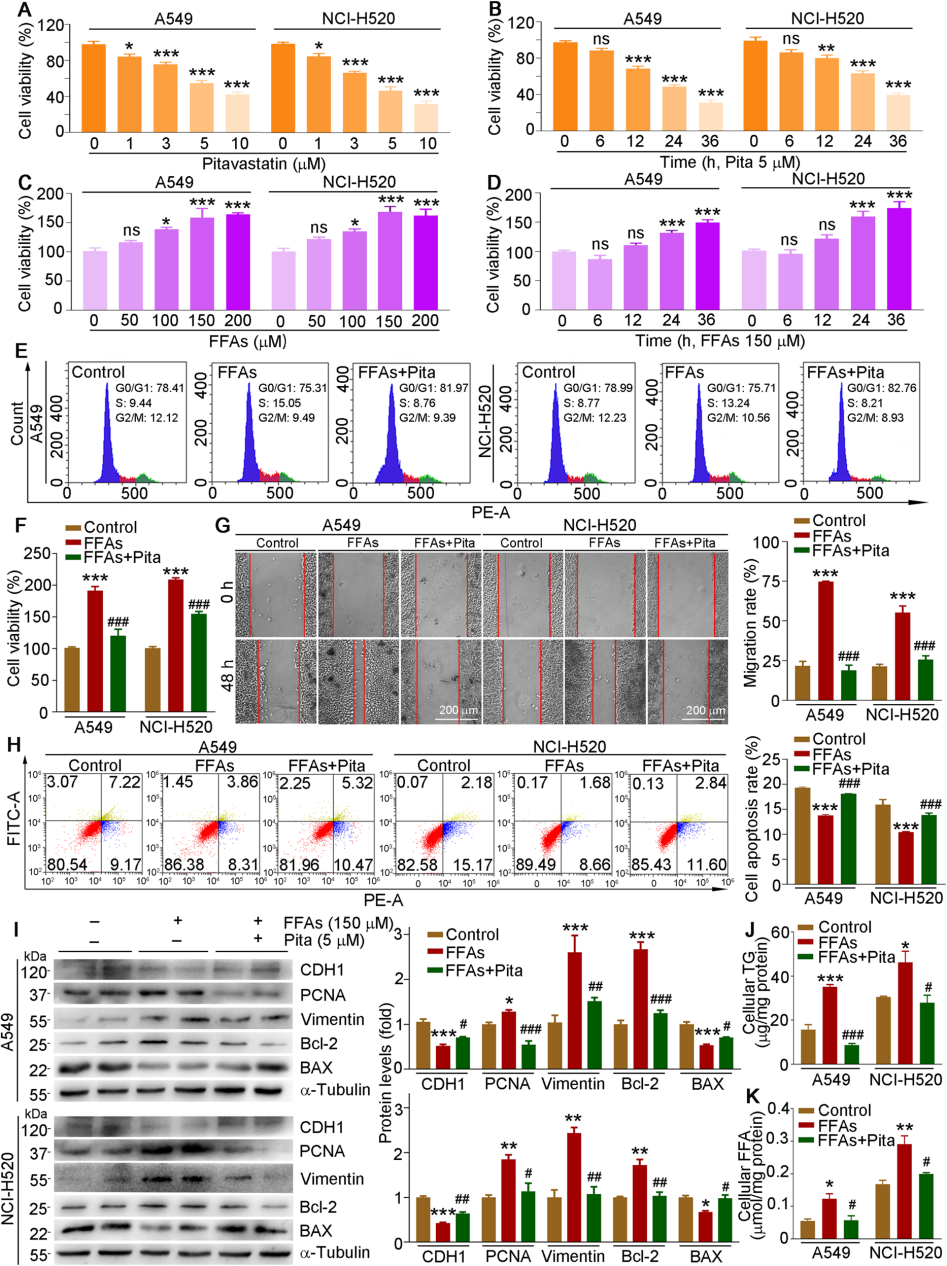
Pitavastatin inhibits FFA-enhanced cell proliferation and migration but enhances apoptosis in lung cancer cell lines. **A–D** A549 and NCI-H520 cells were treated with indicated concentration of pitavastatin (**A**) or FFAs (**C**) for 24 h, or 5 µM pitavastatin (**B**) or 150 µM FFAs (**D**) at the indicated time. Cell viability was determined using MTT assay. **E-K** A549 and NCI-H520 cells are treated with 150 µM FFAs or 5 µM pitavastatin plus 150 µM FFAs for 24 h (others) or 48 h (**G**); FACS (**E**), MTT (**F**), scratch (**G**), and Annexin V-FITC/PI staining (**H**) assays were used to evaluate cell cycle, viability, migration, and apoptosis, respectively. Protein expression of CDH1, PCNA, vimentin, Bcl-2, and BAX was determined by Western blot with density quantitative analysis (**I**). Cellular TG (**J**) and FFA (**K**) levels were measured by indicated assay kits. Mean ± SEM; *p < 0.05; **p < 0.01; ***p < 0.001 vs control group; ^#^*p* < 0.05; ^##^*p* < 0.01; ^###^*p* < 0.001 vs FFA-treated group; **A**–**D** and **F**: *n* = 6; **E**, **G**–**K**: *n* = 3; Pita, pitavastatin

Chronic HFD intake can promote multiple types of tumor progression. However, the role of HFD and lipid-lowering drug, pitavastatin, on lung cancer progression is still unclear. Therefore, we used the mixture solution of palmitate acid (PA) and oleic acid (OA) as exogenous FFAs to mimic a high-fat environment. First, we determined the effect of pitavastatin on proliferation. We treated A549 and NCI-H520 cells with pitavastatin and then determined cell viability by the MTT method. As shown in Fig. 1A, B, pitavastatin reduced cell viability in dose- and time-dependent manners, whereas the cell viability of A549 and NCI-H520 cells was increased by FFAs (Fig. 1C, D). To further explore the role of pitavastatin in FFA-enhanced cell proliferation, we treated the cells with FFAs or pitavastatin plus FFAs and found that pitavastatin largely reduced FFA-enhanced cell proliferation (Fig. 1F). Moreover, the results of cell cycle analysis conducted by flow cytometry indicated that FFAs increased the percentage of cells in S phase, which was attenuated by pitavastatin treatment (Fig. 1E). These data suggest that exogenous lipids promote proliferative capacity by inducing the transition from G1 to S phase. Additionally, the wound healing test indicated that FFAs significantly promoted cell migration, which was completely blocked by pitavastatin in both A549 and NCI-H520 cells (Fig. 1G).

In order to explore the effect of FFAs and pitavastatin on cell apoptosis, we treated cells and measured the proportion of cell apoptosis by Annexin V-FITC/PI staining assay kit. Our results showed that FFAs significantly reduced A549 and NCI-H520 cell apoptosis at both early and late stages, while pitavastatin reversed FFA-inhibited cell apoptosis (Fig. 1H). To unravel the underlying mechanisms, we determined expression of proteins involved in proliferation (proliferating cell nuclear antigen, PCNA), migration and invasion [cadherin 1 (CDH1) and vimentin], and apoptosis [B-cell lymphoma/leukenfia-2 (Bcl-2) and Bcl-2-associated X (BAX)] in A549 and NCI-H520 cells. We found that the protein expression of PCNA, vimentin, and Bcl-2 was increased, but that of CDH1 and BAX was decreased by FFAs, in both A549 and NCI-H520 cells. However, these changes were reversed by pitavastatin administration (Fig. 1I). Taken together, the above results suggest that pitavastatin attenuated FFA-enhanced cell viability, and the effects were associated with the regulation of proteins involved in cell proliferation, migration, invasion, and apoptosis.

Mounting evidence indicates that the excess uptake and production of FFAs in most solid malignancies is increased. The FFAs provide nutrients and energy for tumor growth and migration. Therefore, we determined the cellular TG, FFA, and cholesterol content. Our results showed that the exogenous FFAs increased TG, FFA, and cholesterol content, which was almost reversed by pitavastatin (Fig. 1J, K and Fig. S1), indicating that pitavastatin-inhibited cell viability may be correlated to the reducing lipid accumulation in cells.

### Pitavastatin inhibits HFD-exacerbated NSCLC progression in C57BL/6J mice

**Fig. 2:**
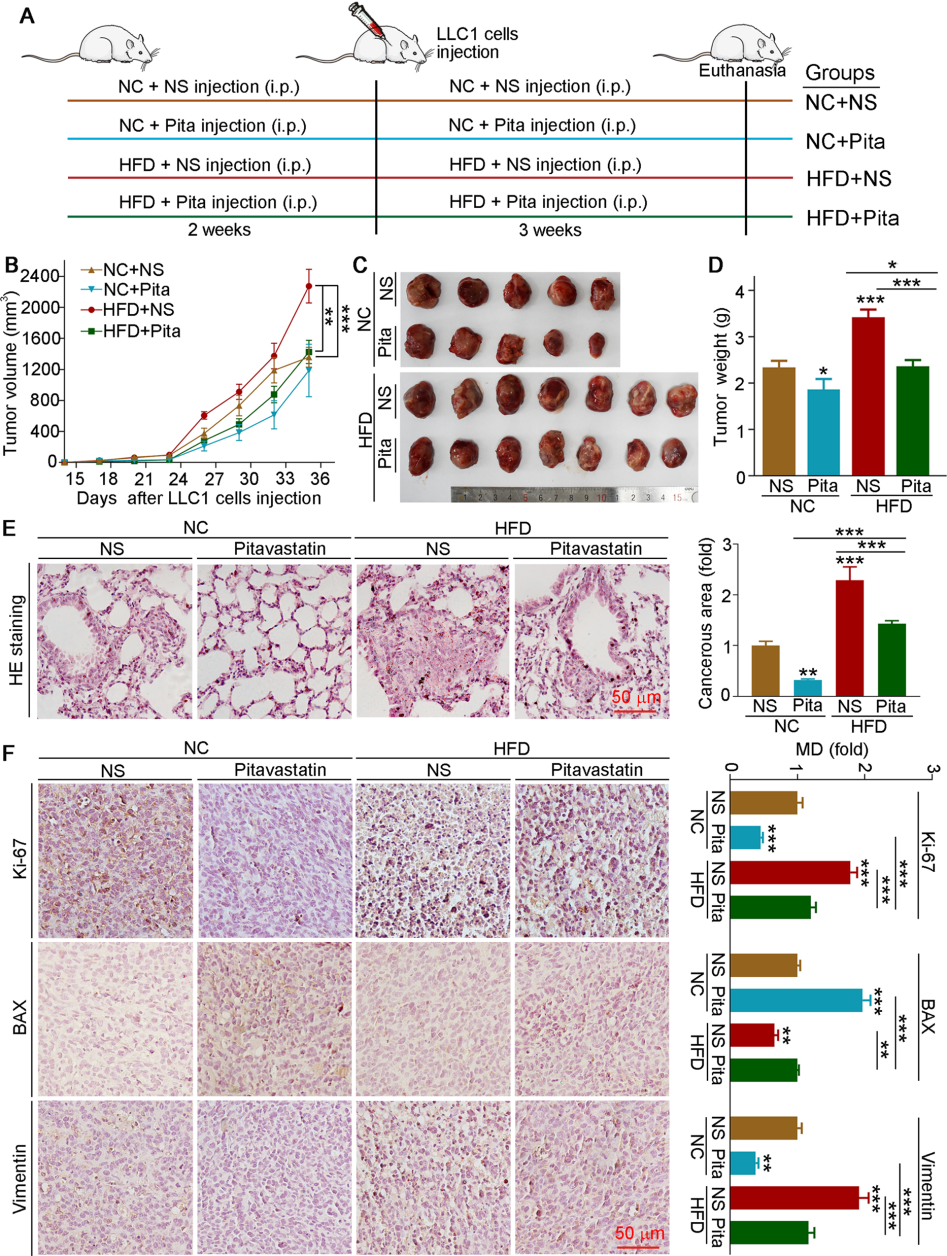
Pitavastatin inhibits the progression of NSCLC in C57BL/6J mice. **A** Experimental design: C57BL/6J mice in four groups received the following treatment: normal chow (NC) groups (5 mice/group), fed normal food and injected with NS or pitavastatin solution (2 mg/kg/day) for 5 weeks; HFD groups (7 mice/group), fed HFD and injected with NS or pitavastatin solution for 5 weeks. At the third week, mice received s.c. injection of LLC1 cells (10^6^ cells/100 µL/mouse). At the end of experiment, blood, tumor, and lung tissues were collected. **B** Tumor size was determined once every 3 days. **C**, **D** Tumor samples were photographed (**C**) and weighted (**D**). **E** Paraffin sections of lung tissues were performed H&E staining, and the lung carcinogenesis area was quantified by ImageJ software. **F** Tumor paraffin sections were performed IHC staining to detect the expression of Ki-67, BAX, and vimentin, and mean density (MD) was quantified by ImageJ software. Mean ± SEM; *p < 0.05; **p < 0.01; ***p < 0.001 (*n* ≥ 5); Pita, pitavastatin

To investigate the role of pitavastatin and HFD on tumor growth in mice, Lewis lung cancer–bearing C57BL/6J mice were fed a normal chow (NC) or HFD in the presence or absence of pitavastatin (Fig. 2A). After euthanasia, mouse tumor tissues were collected. Compared to NC group, we found that the tumor volume and weight were increased in the HFD group mice. Importantly, pitavastatin administration decreased tumor volume and weight in both NC and HFD feeding conditions (Fig. 2B–D). In addition, HFD exacerbated metastasis in lung tissues, which was also attenuated by pitavastatin treatment (Fig. 2E). At the molecular level, tumor tissues of the HFD group mice exhibited high expression of Ki-67 and vimentin, but low expression of BAX. However, pitavastatin treatment significantly increased BAX, while inhibited Ki-67 and vimentin expressions in both feeding conditions (Fig. 2F). Taken together, our results show that HFD promotes tumor growth, which can be largely reversed by the administration of pitavastatin.

Epidemiological evidence suggests that HFD alters lipid composition in the tumor microenvironment, which affects the activation of oncogenes and promotes tumor progression (Peck and Schulze 2019). Subsequently, we measured lipid levels in serum and tumor tissues. We found that HFD elevated serum T-CHO, LDL-C, HDL-C, TG, and FFA levels (Table S2). Consistent with the tumor inhibition effects, pitavastatin also reduced HFD-induced T-CHO, TG, and FFAs levels in serum. Furthermore, we indicated that both TG and FFA levels in orthotopic tumors were enhanced by HFD, and these changes were nearly reversed by the administration of pitavastatin. Therefore, our *in vitro* and *in vivo* results reveal that pitavastatin significantly inhibits tumor progression by regulating lipid metabolism.

### Pitavastatin inhibits lung cancer progression by reducing CD36 expression

**Fig. 3:**
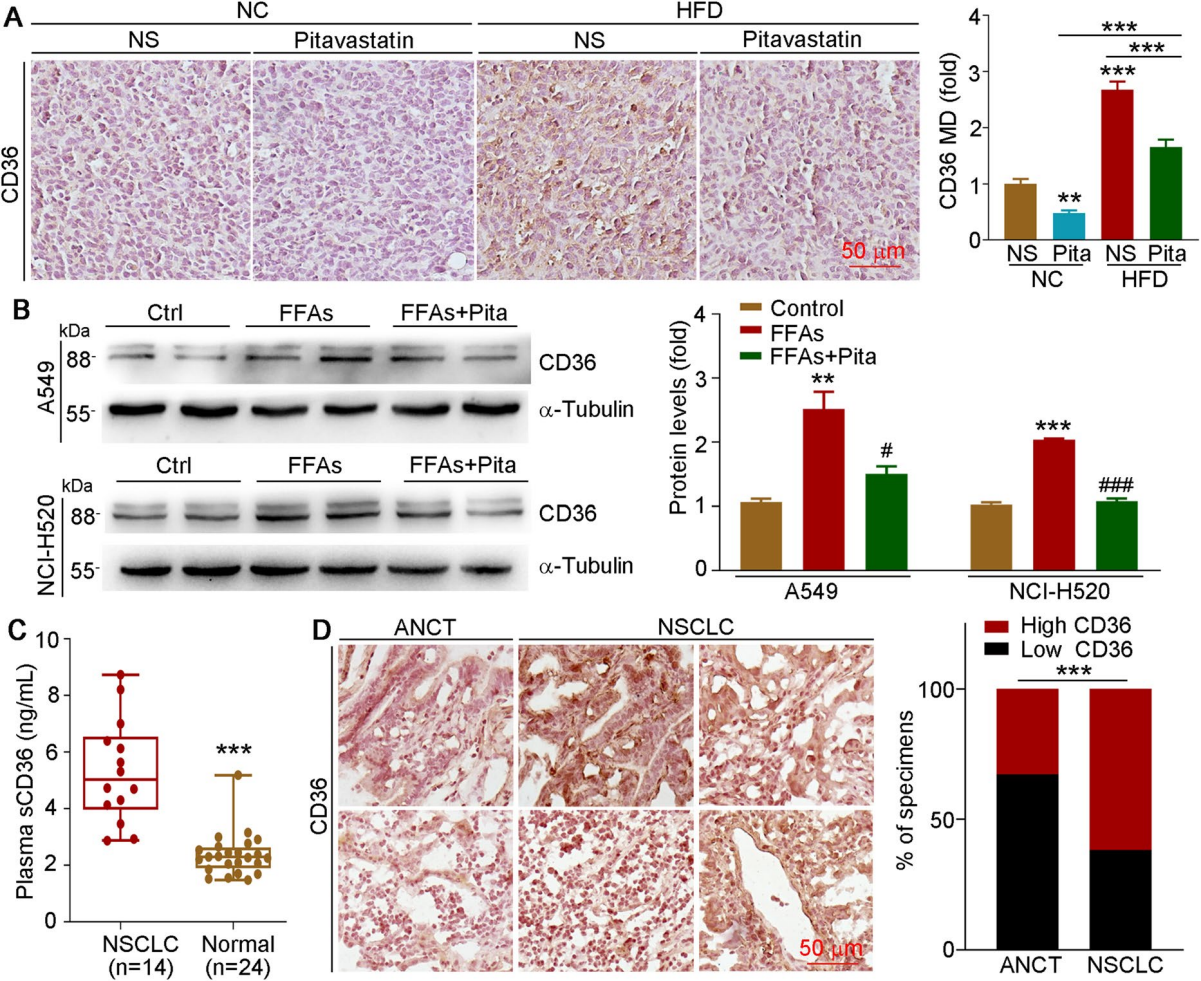
CD36 expression is positively associated with lung cancer development. **A** Tumor paraffin sections collected from Fig. 2A were conducted IHC staining to detect CD36 expression with quantified MD by ImageJ software; mean ± SEM; *p < 0.05; ***p < 0.001 (*n* = 5). **B** A549 and NCI-H520 cells were treated with 150 µM FFAs or 5 µM pitavastatin plus FFAs for 24 h; protein expression of CD36 was determined by Western blot with density quantitative analysis. Mean ± SEM; **p < 0.01; ***p < 0.001 vs control group; ^#^*p* < 0.05; ^###^*p* < 0.001 vs FFA-treated group (*n* = 3); Pita, pitavastatin. **C** Plasma sCD36 levels were detected by the CD36 ELISA kit. Mean ± SEM; ***p < 0.001. **D** Expression of CD36 in cancer tissues (*n* = 5) and adjacent non-cancerous tissues (*n* = 2) was detected by IHC staining, and MD was quantified by ImageJ software. ANCT, adjacent non-cancerous tissues

CD36 is a receptor of fatty acids that can promote the development of cardiovascular diseases and cancer (Wang and Li 2019). To evaluate whether CD36 is involved in HFD-enhanced or pitavastatin-reduced cancer, we determined CD36 expression in tumor samples or cells. As shown in Fig. 3A, we found that CD36 levels in tumors of the HFD groups were higher than those in the tumors of the NC groups, while pitavastatin inhibited CD36 expression in those two conditions. Consistently, the *in vitro* results also found that CD36 expression was enhanced by FFAs in A549 and NCI-H520 cells but reduced by pitavastatin treatment (Fig. 3B). In order to explore whether CD36 is related to the development of NSCLC, we collected serum from 14 NSCLC patients and 24 healthy human volunteers. Our results found that soluble CD36 (sCD36) levels in plasma were higher in NSCLC patients than in healthy ones (Fig. 3C). More importantly, we showed that CD36 protein levels in tumor tissues of NSCLC patients were much higher than those in adjacent non-cancerous tissues (Fig. 3D). To sum up, the above results suggest that CD36 may play an important role in NSCLC progression.

**Fig. 4:**
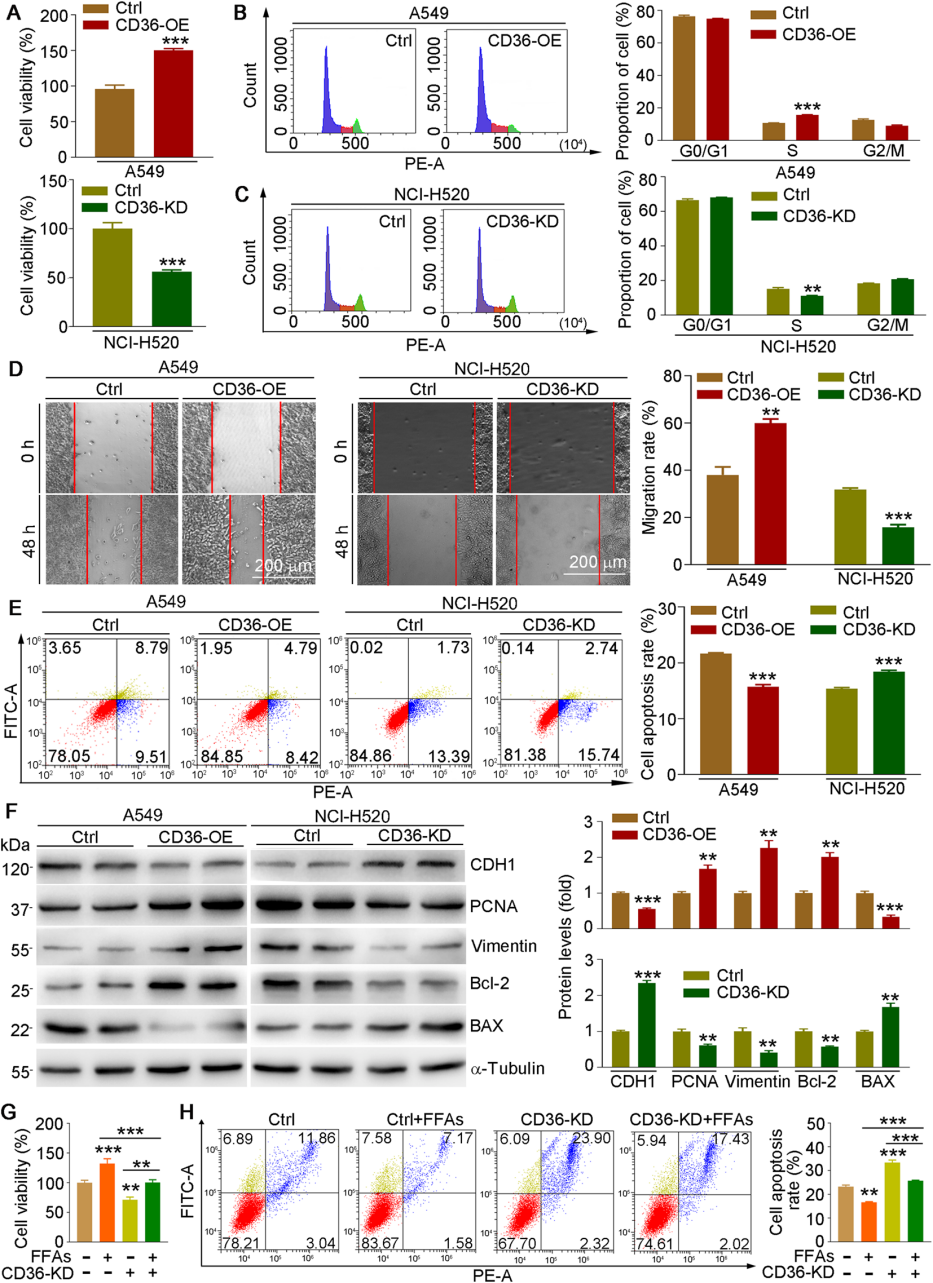
CD36 expression is positively correlated with cell proliferation and migration *in vitro*. **A–F** A549 cells were transfected with pCMV or pCMV-CD36 plasmid, and NCI-H520 cells were transfected with CasRX or CasRX-CD36 plasmid for 12 h in serum-free medium and then cultured in complete medium for another 24 h. Cells were collected for determination of cell viability (**A**), cell cycle (**B**, **C**), migration (**D**), and apoptosis (**E**) by MTT assay, FACS, wound healing test, and Annexin V-FITC/PI staining, respectively. Protein expression of CD36, CDH1, PCNA, vimentin, Bcl-2, and BAX was determined by Western blot with density quantitative analysis (**F**). **G**, **H** NCI-H520 cells were transfected with CasRX or CasRX-CD36 plasmid for 12 h and then cultured in complete medium for 24 h, followed by FFAs (150 µM) treatment for 24 h. Cells were collected for determination of cell viability (**G**) and apoptosis (**H**). Mean ± SEM; *p < 0.05; **p < 0.01; ***p < 0.001; **A**, **G**: *n* = 6; **B**–**F**, **H**: *n* = 3

To further investigate the critical role of CD36 in NSCLC, we transfected A549 cells with the pCMV-CD36 plasmid for overexpression or NCI-H520 cells with the CasRX-CD36 plasmid to knockdown CD36. First, we verified the transfection efficacy of plasmids, and CD36 levels were enhanced ~ 2.5- or ~ 0.4-fold by transfection with the pCMV-CD36 or CasRX-CD36 plasmid, respectively (Figure S2). CD36 overexpression enhanced cell viability (Fig. 4A, upper panel), proliferation (Fig. 4B), and migration (Fig. 4D, left and right panels) while reducing the ratio of apoptotic cells (Fig. 4E, left and right panels). In contrast, cell viability (Fig. 4A, lower panel), proliferation (Fig. 4C), and migration (Fig. 4D, middle and right panels) were reduced in CD36-knockdown cells, but cell apoptosis was enhanced (Fig. 4E, middle and right panels). More importantly, at the molecular level, cells overexpressing CD36 showed enhanced PCNA, vimentin, and Bcl-2 expressions but reduced CDH1 and BAX levels (Fig. 4F, left and right panels). Consistent with cell phenotype results, proliferation-, migration- and apoptosis-related protein expressions were also regulated by CD36 inhibition, which was opposite to observations in the overexpressed cells (Fig. 4F, middle and right panels). Moreover, FFA-enhanced cell viability was largely reduced (Fig. 4G), while the reduction effects of FFA on cell apoptosis were enhanced (Fig. 4H) in CD36-knockdown NCI-H520 cells. Consistently, FFA-regulated vimentin, PCNA, and BAX were also attenuated in CD36-knockdown NCI-H520 cells (Figure S3). Taken together, these results indicate that CD36 levels are positively associated with cancer development.

**Fig. 5:**
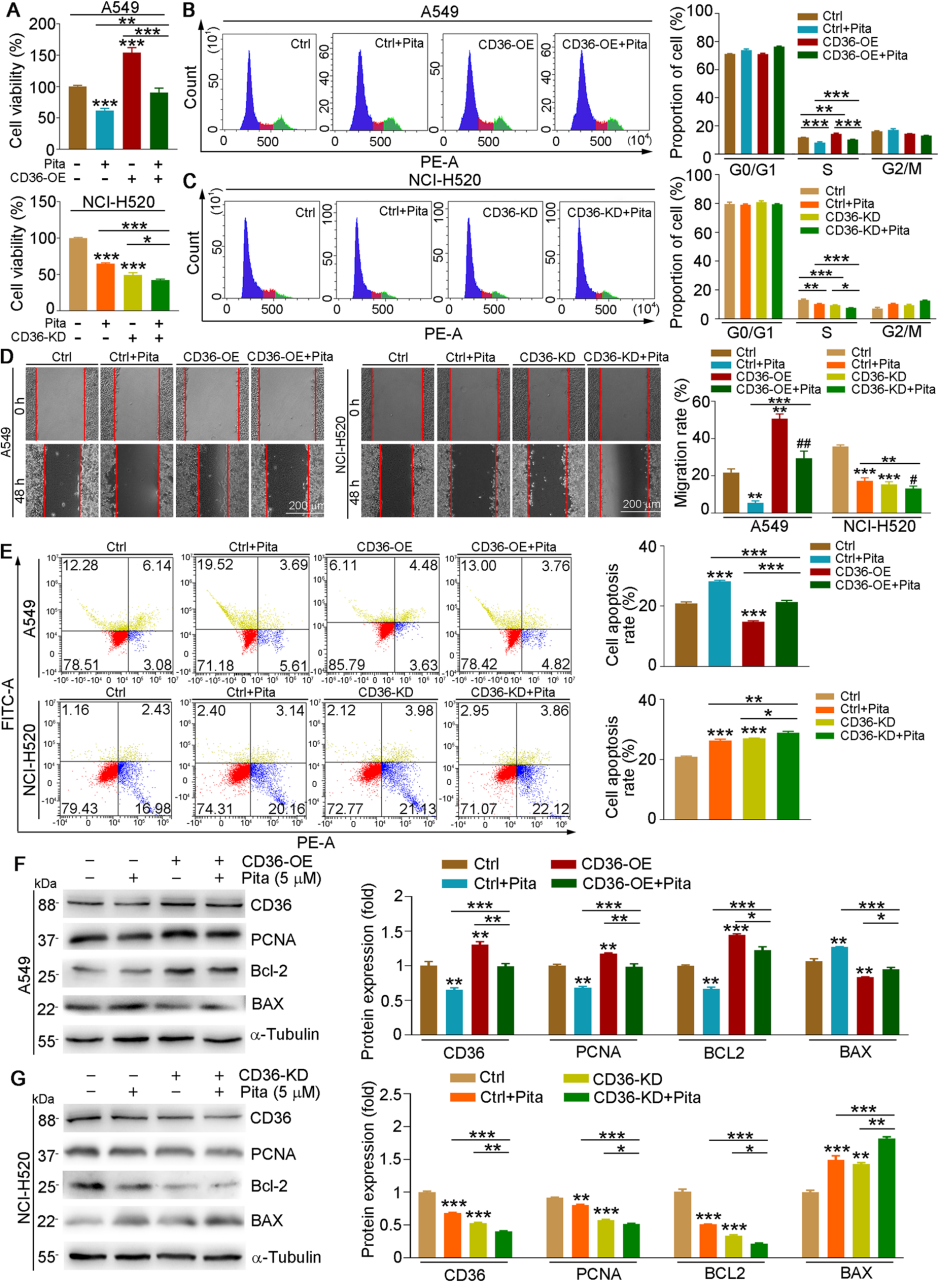
The effects of pitavastatin on cell proliferation, migration, and apoptosis are related to CD36 expression. A549 cells were transfected with pCMV or pCMV-CD36 plasmid, and NCI-H520 cells were transfected with CasRX or CasRX-CD36 plasmid for 12 h and then cultured in complete medium for 24 h, followed by received pitavastatin (5 µM) treatment for 24 h. Cells were collected for determination of cell viability (**A**), cell cycle (**B**, **C**), migration (**D**), and apoptosis (**E**). Protein expression of CD36, PCNA, Bcl-2, and BAX was determined by Western blot with density quantitative analysis (**F**, **G**). Mean ± SEM; *p < 0.05; **p < 0.01; ***p < 0.001; **A**: *n* = 6; **B**–**F**: *n* = 3; Pita, pitavastatin

The results in Fig. 3A, B showed that pitavastatin reduced HFD or FFA-induced CD36 expression in tumor tissues or cells, indicating that pitavastatin-reduced cancer development may be regulated by CD36. Therefore, we treated CD36-knockdown or overexpression cells with or without pitavastatin. Our results showed that CD36 overexpression enhanced cell viability (Fig. 5A, upper panel), proliferation (Fig. 5B), and migration (Fig. 5D, left and right panels) but reduced cell apoptosis (Fig. 5E, upper panel), which was largely attenuated by pitavastatin in A549 cells. In contrast to CD36 overexpression, both CD36 knockdown and pitavastatin treatment inhibited cell viability (Fig. 5A, lower panel), proliferation (Fig. 5C), and migration (Fig. 5D, middle and right panels) but increased cell apoptosis (Fig. 5E, lower panel) in NCI-H520 cells. Consistent with the phenotypic results in cells, pitavastatin also reversed CD36 overexpression–regulated cell proliferation-, migration-, and apoptosis-related protein expression in A549 cells (Fig. 5F). However, the regulatory effect of pitavastatin in related protein expression was attenuated in CD36-knockdown cells (Fig. 5G). Therefore, we demonstrate that CD36 is crucial to the development of NSCLC and that pitavastatin-mediated regulation of cancer processes depends on CD36 expression, at least in part.

### The inhibitory effects of pitavastatin on HFD-enhanced tumor progression are impaired in CD36^−/−^ mice

**Fig. 6:**
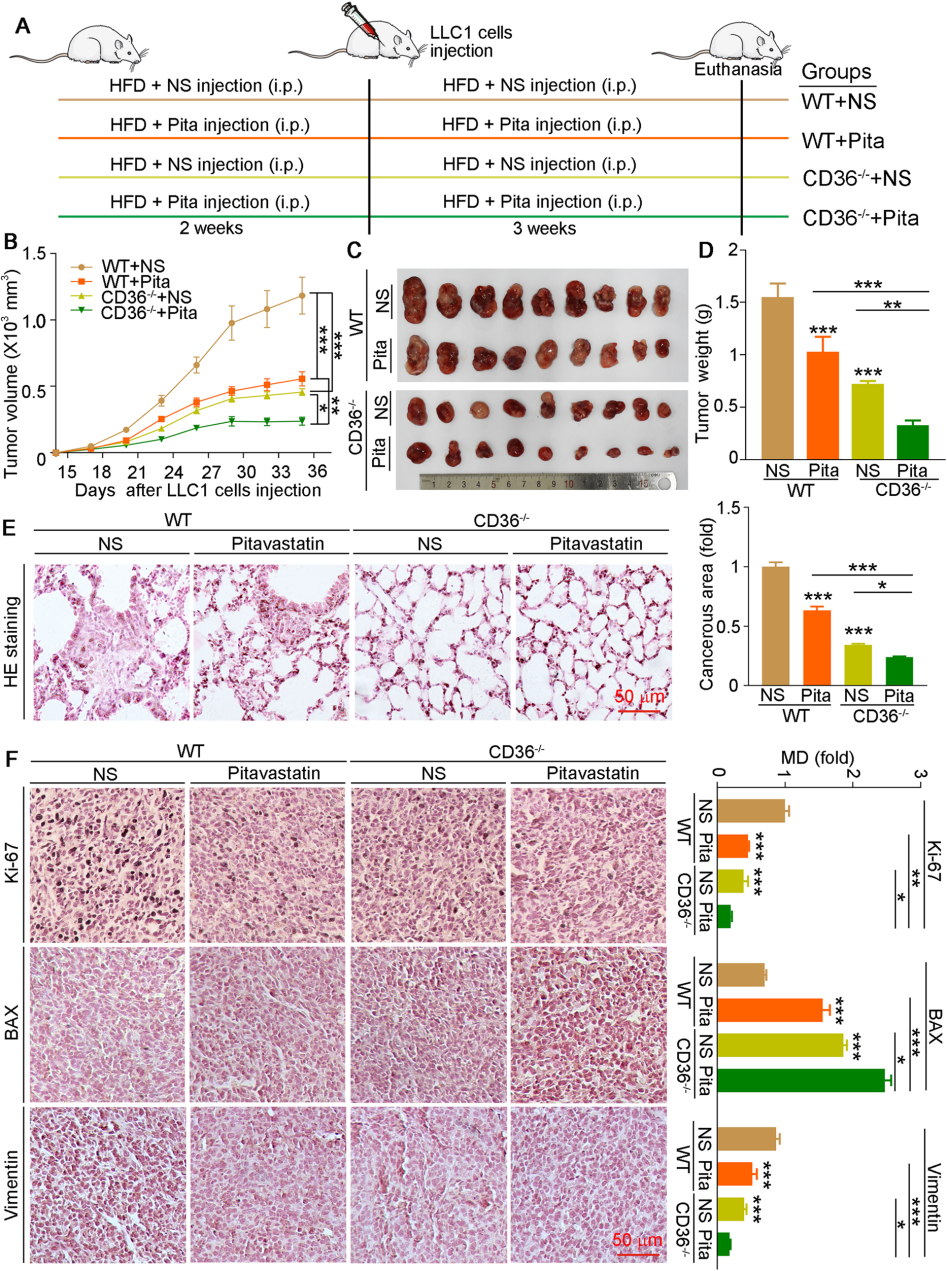
The reduction effects of pitavastatin on tumor progression were impaired in CD36^−/−^ mice. **A** Experimental design: C57BL/6J and CD36^−/−^ mice (9 mice/group) in four groups fed with HFD and injected with NS or pitavastatin solution (2 mg/kg/day) for 5 weeks. At the third week, mice received s.c. injection of LLC1 cells (10^6^ cells/100 µL/mouse). At the end of experiment, blood, tumor, and lung tissues were collected. **B** Tumor size was determined once every 3 days. **C**, **D** Tumor tissues were photographed (**C**) and weighted (**D**). **E**, **F** Paraffin sections of lung tissues were performed H&E staining, and lung carcinogenesis area was quantified by ImageJ software (**E**), or conduced IHC staining to detect Ki-67, BAX, and vimentin expression with MD quantified by ImageJ software (**F**). Mean ± SEM; *p < 0.05; **p < 0.01; ***p < 0.001; **B**, **D**: *n* = 8; **E**, **F**: *n* = 5; Pita, pitavastatin

The above *in vitro* results indicated that pitavastatin-reduced cell proliferation almost depends on CD36 reduction. Therefore, we used global CD36-deficient (CD36^−/−^) mice for determining the protective role of pitavastatin in lung cancer. C57BL/6J and CD36^−/−^ mice were fed with HFD and received normal saline (NS) or pitavastatin injection for 5 weeks. At the third week, mice were injected with LLC1 cells (Fig. 6A). Compared to C57BL/6 J mice, tumor progression was largely impaired in CD36^−/−^ mice. In addition, pitavastatin administration reduced tumor size and weight in both C57BL/6J and CD36^−/−^ mice. However, the protective effects of pitavastatin in CD36^−/−^ mice were weaker than those in C57BL/6 J mice (Fig. 6B–D). In addition, H&E staining showed that tumor metastasis was suppressed in the lung tissues of CD36^−/−^ mice (Fig. 6E). Furthermore, the results of IHC staining of tumor sections showed that the expression of Ki-67 and vimentin in CD36^−/−^ mice was much lower than that in C57BL/6J mice, while BAX expression was higher in CD36^−/−^ mice (Fig. 6F). Consistent with the tumor reduction effects, pitavastatin also regulated related protein expression but exhibited fewer effects in CD36^−/−^ mice. Our data suggest that CD36 deficiency greatly reduces lung cancer progression and that the protective effect of pitavastatin on lung cancer is impaired in CD36^−/−^ mice.

We indicated that pitavastatin inhibited cell proliferation and cancer development through regulating lipid profiles (Fig. 1J, K and Table S2). To explore whether the impaired protective role of pitavastatin in CD36^−/−^ mouse is also related to lipid levels, we determined lipid profiles in serum and tumor tissues. As shown in Table S3, we found that lipid levels in CD36^−/−^ mouse serum were much lower than those in WT mouse serum. In addition, the reduction effects of pitavastatin in T-CHO, TG, and FFA levels were largely impaired in CD36^−/−^ mice. Taken together, these results indicate that pitavastatin-mediated reduction in tumor progression is partly dependent on CD36 expression and lipid levels.

### Pitavastatin-attenuated tumor progression is regulated by the CD36/AKT/mTOR pathway

**Fig. 7:**
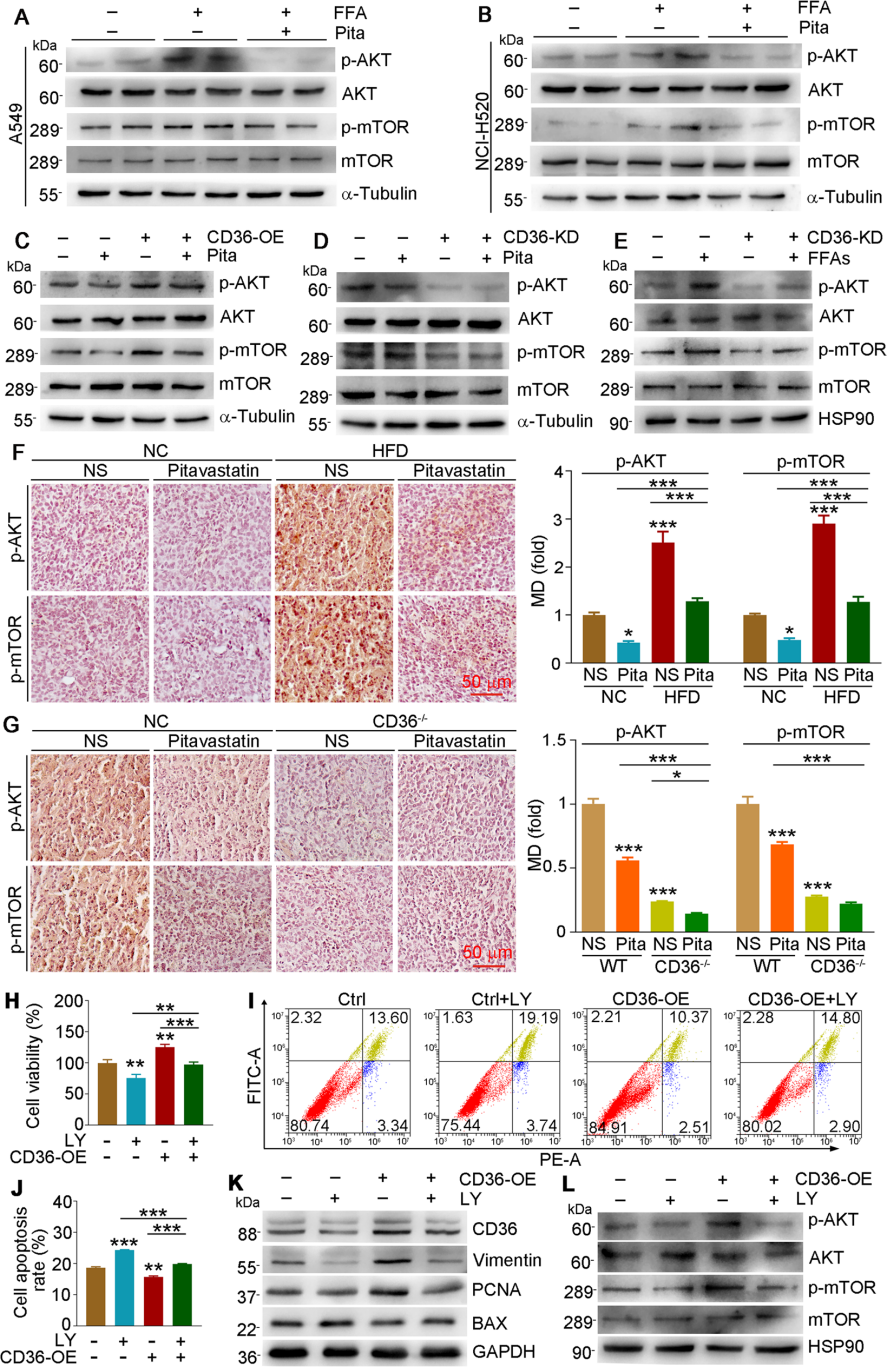
The reduction effects of pitavastatin on tumor progression are regulated by CD36/AKT/mTOR pathway. **A–E** A549 (**A**) and NCI-H520 (**B**) cells were treated with 150 µM FFAs or 5 µM pitavastatin plus FFAs for 24 h. A549 cells were transfected with pCMV or pCMV-CD36 plasmid for 12 h (**C**); NCI-H520 cells were transfected with CasRX or CasRX-CD36 plasmid (**D**, **E**) for 12 h and then cultured in complete medium for 24 h, followed by treatment with 5 µM pitavastatin (**C**, **D**) or 150 µM FFAs (**E**) for 24 h. Protein expression of p-AKT, AKT, p-mTOR, and mTOR was detected by Western blot. **F**, **G** Tumor paraffin sections collected from Fig. 2A (**F**) or Fig. 6A (**G**) were performed IHC staining to detect the expression of p-AKT and p-mTOR with MD quantified by ImageJ software. **H–L** A549 cells were transfected with pCMV or pCMV-CD36 plasmid for 12 h and then cultured in complete medium for 24 h, followed by received LY294002 (10 µM) treatment for 24 h. Cells were collected for determination of cell viability (**H**) and apoptosis (**I**, **J**). Protein expression of CD36, vimentin, PCNA, BAX, p-AKT, AKT, p-mTOR, and mTOR was determined by Western blot (**K**, **L**). Mean ± SEM; *p < 0.05; **p < 0.01; ***p < 0.001; **A**–**E**, **I**–**L**: *n* = 3; **F**, **G**: *n* = 5; **H**: *n* = 6; Pita, pitavastatin; LY, LY294002

The AKT-mTOR pathway is activated in varieties of tumors and performs an important function in regulating cell growth, promoting cell invasion and metastasis, and enhancing neo-angiogenesis (LoRusso 2016). To explore whether the AKT-mTOR pathway is involved in pitavastatin-reduced or CD36-induced cell proliferation, we determined the activation of AKT and mTOR in cells and tumor tissues. Both phosphorylated AKT (p-AKT) and p-mTOR levels were enhanced by exogenous FFA treatment while being significantly reduced by pitavastatin treatment in A549 and NCI-H520 cells (Fig. 7A, B and Fig. S4A and B). In addition, we found that CD36 overexpression also activated AKT and mTOR (Fig. 7C and Fig. S4C). In contrast, reduction of CD36 expression inhibited p-AKT and p-mTOR levels (Fig. 7D and Fig. S4D). Either reduced or enhanced p-AKT and p-mTOR by CD36 inhibition or overexpression can also be further regulated by pitavastatin. Compared to normal or overexpression cells, the reduction effects of pitavastatin on the levels of p-AKT and p-mTOR were lower in CD36-knockdown cells. Notably, compared with control cells, the upregulated levels of p-AKT and p-mTOR by exogenous FFAs in CD36-knockdown NCI-H520 cells were obviously impaired (Fig. 7E and Fig. S4E). Moreover, *in vivo* results also indicated that pitavastatin reduced p-AKT and p-mTOR levels in tumor tissues of both NC and HFD conditions (Fig. 7F, G). Consistent with *in vitro* results (Fig. 7D), we found that the reduction effect of pitavastatin on p-AKT and p-mTOR expressions in tumor tissues of CD36^−/−^ mice was less than that in WT mice (Fig. 7G).

To further confirm the association between CD36 and the AKT pathway, we treated A549 cells with LY294002 (an AKT inhibitor) and found that LY294002 significantly inhibited cell viability (Fig. 7H) but enhanced apoptosis (Fig. 7I, J), with greater degree in CD36-overexpressed A549 cells than in control cells. In addition, LY294002 also regulated related protein levels, and the activity of AKT and mTOR (Fig. 7K, L and Fig. S4F and G). Therefore, our results suggest that pitavastatin attenuates HFD-accelerated NSCLC development contributed by reducing CD36-AKT-mTOR pathway, at least in part.

## Discussion

Diet is a major contributing factor to the progression of cancers worldwide (Peck and Schulze 2019). In recent years, studies have demonstrated that a HFD has an accelerating effect on a variety of types of cancer (Narita et al. 2019). In this study, we found that exogenous FFAs or HFD promoted cell proliferation or tumor progression (Figs. 1 and 2). Along with the enhanced lipid profiles, CD36 expression was also enhanced by FFAs or HFD. Additionally, CD36 expression was found to be elevated in the plasma and tumor tissue of NSCLC patients (Fig. 3). Furthermore, CD36 inhibition by pitavastatin or knockdown/knockout strategies significantly reduced cell viability and tumor progression (Figs. 4, 5 and 6). Consequently, we clearly demonstrate that inhibition of CD36 protein expression can mitigate HFD-accelerated lung cancer. Accumulating evidence suggests that lipid metabolism tends to play important roles in different stages of cancer (Martin-Perez et al. 2022; Peck and Schulze 2019). Excess lipid intake or metabolic syndrome leads to dysregulation of lipid metabolism, predisposing tumor initiation and development. In addition, the increasing amount of fat inhibits immune monitoring and overall favors cancer development (Kulkarni and Bowers 2021). Fatty acids can promote cell proliferation, migration, and angiogenesis (Pascual et al. 2021; Rose and Connolly 2000). In addition, enhanced fatty acid oxidation provides energy for facilitating cancer development by regulating cell transformation and tumorigenesis signaling pathways (Currie et al. 2013). In this study, we indicated that exogenous FFAs have significant enhancement effects on cell proliferation and migration in both A549 and NCI-H520 cells (Fig. 1). In addition, the *in vivo* results demonstrated that HFD significantly increased the volume and weight of tumors (Fig. 2). Conversely, both *in vitro* and *in vivo* results showed that pitavastatin substantially inhibited cell viability and tumor progression (Figs. 1 and 2). In line with these findings, serum and tumor lipid profiles were also enhanced by FFAs or HFD (Fig. 1J, K and Table S2). Therefore, our results indicate that NSCLC progression is positively related to lipid levels.

CD36, identified as a receptor for fatty acids, has pivotal functions in both metabolism and immunity (Ma et al. 2021; Yang et al. 2016). Transcription of CD36 can be regulated by multiple transcription factors and ligands. CD36 is a downstream target of PPARγ. Components of ox-LDL and ox-LDL can activate PPARγ and subsequently induce CD36 expression. Natural or synthetic PPARγ ligands, such as lipids, induce CD36 expression and then stimulate additional lipid deposition (Lee and Evans 2002). In addition, our previous study has found that reactive oxygen species generation induced CD36 expression by enhancing translational efficiency (Yang et al. 2015). On the other hand, the expression of CD36 can be modified in a post-translational manner. Including glycosylation, phosphorylation, ubiquitylation, and palmitoylation (Zhang et al. 2021). In mice fed with high-fat and high-cholesterol diet, CD36 stability was enhanced by monoubiquitination by an E3 ubiquitin ligase, which was contrary to the common fact that ubiquitinated proteins are degraded by the proteasome (Kim et al. 2011). In another study, the authors showed that in gastric cancer, fatty acid induced CD36 expression through O-GlcNAcylation (Jiang et al. 2019). Taken together, FFA or HFD regulates CD36 via multiple pathways in different situations. In this study, we found that the protein expression of CD36 was increased by the administration of FFAs and HFD (Fig. 3A, B). However, pitavastatin had little effect on PPARγ expression in this study (data not shown), and the specific mechanisms underlying how FFAs or HFD regulates CD36 need further investigation. In recent years, it has been gradually proposed that CD36 plays a key role in multiple types of cancer. CD36-driven fatty acid metabolism is indispensable in the development of cancer. CD36 is highly expressed in metastatic ovarian tumors, reprogramming tumor metabolism to maintain rapid tumor growth and metastasis (Ladanyi et al. 2018). In gastric cancer, CD36 is a key receptor for PA to induce cancer cell metastasis (Pan et al. 2019). In our study, we not only found that CD36 protein expression was increased by FFAs or HFD (Fig. 3A, B). More importantly, sCD36 levels in plasma were higher in NSCLC patients than in healthy ones (Fig. 3C). In addition, CD36 expression in the tumor tissues of NSCLC patients was much higher than that in adjacent tissues (Fig. 3D). Further study revealed that cell proliferation and tumor progression were increased in cell overexpression CD36 while being reduced by CD36 inhibition (Figs. 4, 5 and 6), indicating that CD36 is a central regulator for NSCLC progression.

Pitavastatin is a potent lipid-lowering drug widely used in the clinic for hypertriglyceridemia. It has also been reported that pitavastatin performs a certain inhibitory effect on tumors (Tilija Pun et al. 2022; Wang et al. 2019). Previous studies have shown that pitavastatin can inhibit multiple cancers, such as liver cancer, breast cancer, colon carcinogenesis, and oral and esophageal squamous cell carcinoma (Wang et al. 2019; Xu et al. 2021; Yasui et al. 2007; You et al. 2016). Moreover, pitavastatin inhibits cancer development via different pathways. In oral and esophageal cancer, it reduces cancer cell growth through suppressing MET signaling by regulating geranylgeranyl diphosphate synthase 1 expression (Xu et al. 2021). In breast cancer, pitavastatin reduced the mevalonate and PPARγ signaling pathways, leading to partial reversal of epithelial-mesenchymal transition by inhibiting Snail and matrix metalloproteinase 9 (Wang et al. 2019). Therefore, pitavastatin reduces cancer development through multiple pathways. In this study, we showed that CD36 plays an important role in NSCLC, but it is not the sole molecular responsible for the inhibitory role of pitavastatin in NSCLS.

The AKT-mTOR signaling pathway is activated in varieties cancers, contributing to cell survival and proliferation (LoRusso 2016). In recent years, small molecule inhibitors targeting AKT and mTOR have been developed for cancer treatment. In previous studies, it has been demonstrated that CD36-mediated cancer is associated with AKT pathway activation, as observed in gastric cancer and hepatic carcinoma (Luo et al. 2021; Pan et al. 2019). It was shown that the Src/PI3K/AKT axis is involved in CD36-mediated HCC and lung cancer development (Liu et al. 2023; Luo et al. 2021). In this current study, we also indicated that the AKT-mTOR pathway was activated by FFAs or HFD, which was associated with the enhanced expression of CD36. However, the inhibition of CD36 expression, either by pitavastatin or gene strategies, reduced the AKT-mTOR pathway (Fig. 7). Moreover, AKT inhibitor can also regulate cell viability and apoptosis in both control and CD36-overexpressed A549 cells (Fig. 7H–J). Taken together, the above results suggest that the AKT-mTOR pathway plays an important role in CD36-mediated lung cancer, at least in part.

In conclusion, this study unveils that a HFD may favor lung cancer initiation, which is contributed by the high lipid levels. Additionally, we identified the enhancing effect of CD36 on NSCLC, which is contributed by the regulation of lipid metabolism and the AKT-mTOR pathway, indicating CD36 may be a novel target for NSCLC treatment. Therefore, inhibition of CD36 by inhibitors may be a promising therapeutic strategy for NSCLC.

## Supplementary information

Below is the link to the electronic supplementary material.

Supplementary file1 (DOC 438 KB)

Supplementary file2 (DOC 67 KB)

Supplementary file3 (DOC 1270 KB)

## Data Availability

All data are available upon request.
