# Supplementary material for: CD36 inhibition reduces non-small-cell lung cancer development through AKT-mTOR pathway
Source: Cell Biol Toxicol. 2024 Feb 6;40(1):10. doi: 10.1007/s10565-024-09848-7 (PMC10847192; doi:10.1007/s10565-024-09848-7)
Supplement: Supplementary file 1 — Supplementary file1 (DOC 438 KB) [file 10565_2024_9848_MOESM1_ESM.doc]

**CD36 inhibition reduces non-small cell lung cancer development through AKT-mTOR pathway**

**
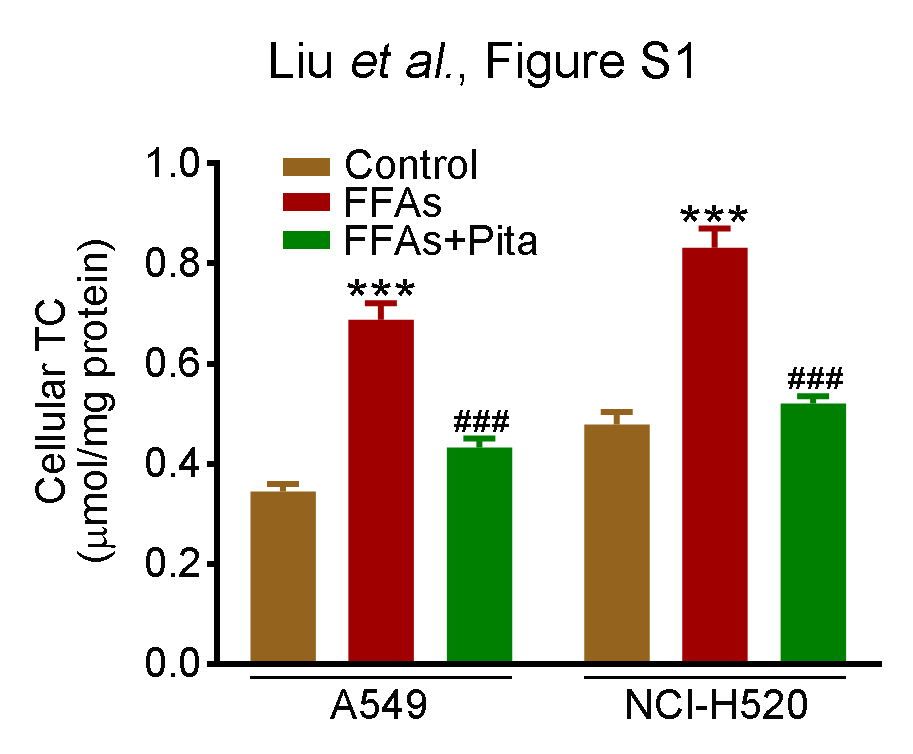
**

**Fig S1. The efficiency of CD36 overexpression or knockdown in cells**

A549 and NCI-H520 cells are treated with 150 μM FFAs or 5 μM pitavastatin plus 150 μM FFAs for 24 h, cholesterol levels were measured by indicated assay kits. mean ± SEM; ***p < 0.001 vs control group; ^###^p < 0.001 vs FFAs-treated group; n = 3; Pita: pitavastatin.


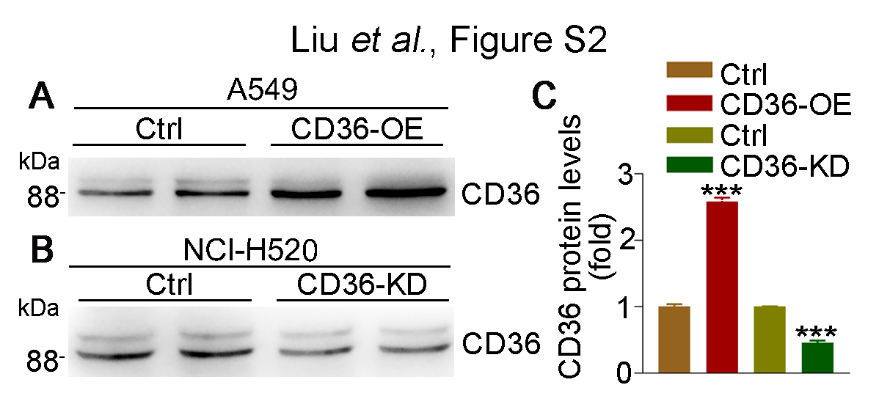


**Fig S2. The efficiency of CD36 overexpression or knockdown in cells**

A549 cells were transfected with pCMV or pCMV-CD36 plasmid, and NCI-H520 cells were transfected with CasRX or CasRX-CD36 plasmid for 12 h in serum-free medium, then cultured in complete medium for another 24 h. Protein expression of CD36 was determined by Western blot (A and B) with density quantitative analysis (C). mean ± SEM; ***p < 0.001; n = 3.

**
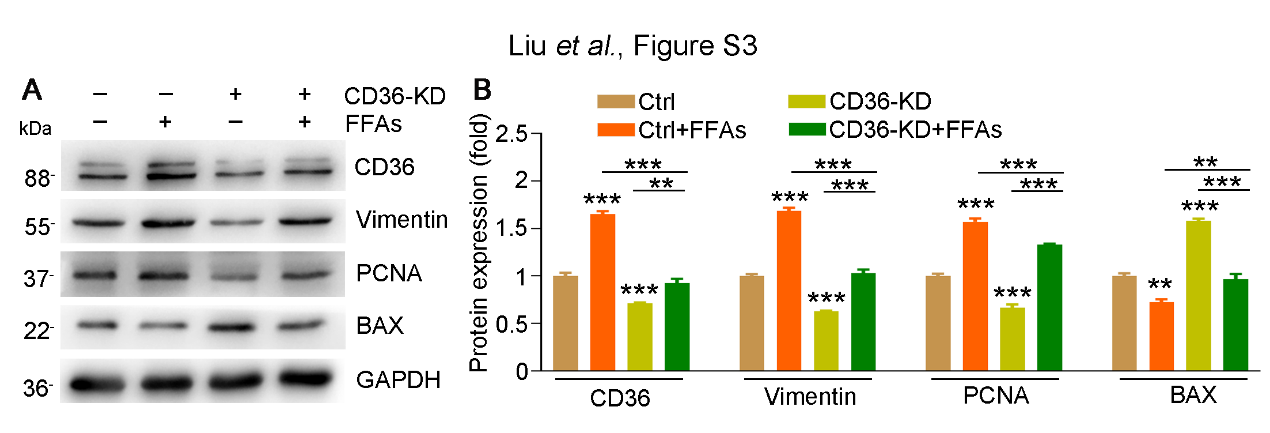
**

**Fig S3.** **The effects of FFAs on cell proliferation-related genes expression is associated with CD36 expression**

NCI-H520 cells were transfected with CasRX or CasRX-CD36 plasmid for 12 h, then cultured in complete medium for 24 h, followed by received FFAs (150 μM) treatment for 24 h. Protein expression of CD36, vimentin, PCNA and BAX was determined by Western blot with density quantitative analysis. mean ± SEM; **p < 0.01; ***p < 0.001; n = 3.

**
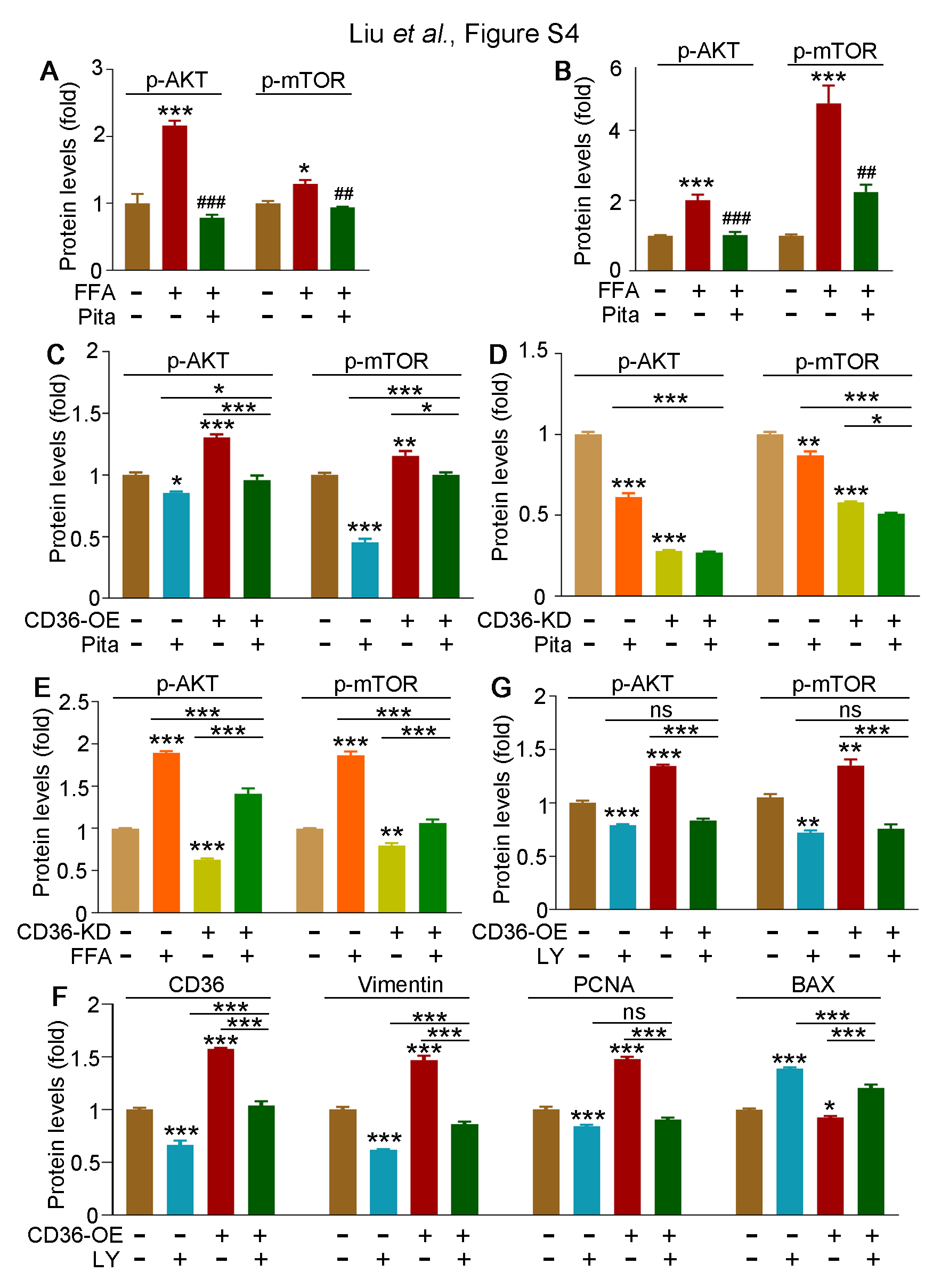
**

**Fig S4. Density quantitative analysis of Western blot in Figure 7**

A-F, the density quantitative analysis of Figure 7A-E, K and L. mean ± SEM; *p < 0.05; **p < 0.01; ***p < 0.001; n = 3; Pita: pitavastatin; LY: LY294002.
