# Supplementary material for: CD36 inhibition reduces non-small-cell lung cancer development through AKT-mTOR pathway
Source: Cell Biol Toxicol. 2024 Feb 6;40(1):10. doi: 10.1007/s10565-024-09848-7 (PMC10847192; doi:10.1007/s10565-024-09848-7)
Supplement: Supplementary file 2 — Supplementary file2 (DOC 67 KB) [file 10565_2024_9848_MOESM2_ESM.doc]

**CD36 inhibition reduces non-small cell lung cancer development through AKT-mTOR pathway**

**Table S1 Antibody information**

| **Antibody** | **Source** | **Catalog#** | **Application (dilution)** |
| --- | --- | --- | --- |
| CD36 | Proteintech | 18836-1-AP | IHC (1:200)  WB (1:2 000) |
| CDH1 | HuaAn  Biotechnology | ET-1607-75 | WB (1:1 000) |
| PCNA | Proteintech | 10205-2-AP | WB (1:2 000) |
| Vimentin | Proteintech | 10366-1-AP | IHC (1:200)  WB (1:2 000) |
| Bcl-2 | Proteintech | 26593-1-AP | WB (1:2 000) |
| BAX | Proteintech | 50599-2-Ig | WB (1:2 000) |
| Ki-67 | Proteintech | 27309-1-AP | IHC (1:2 000) |
| AKT | Proteintech | 10176-2-AP | WB (1:2 000) |
| p-AKT (Ser473) | Proteintech | 80455-1-RR | IHC (1:200)  WB (1:2 000) |
| mTOR | Proteintech | 28273-1-AP | WB (1:2 000) |
| p-mTOR (Ser 2448) | HuaAn  Biotechnology | HA600094 | IHC (1:200)  WB (1:1 000) |
| α-Tubulin | Proteintech | HRP-66031 | WB (1:10 000) |

**Table S2 Pitavastatin improves HFD-induced lipids in C57BL/6J mice**

| Groups | NC | | HFD | |
| --- | --- | --- | --- | --- |
|  | NS | Pita | NS | Pita |
| Serum TG (mM) | 1.06 ± 0.08 | 0.64 ± 0.11** | 1.64 ± 0.18*** | 1.18 ± 0.12^##,&&&^ |
| Serum FFA (U/L) | 1.54 ± 0.07 | 1.03 ± 0.08** | 2.15 ± 0.17*** | 1.54 ± 0.23^###,&&&^ |
| T-CHO (mM) | 1.34 ± 0.08 | 0.88 ± 0.14*** | 1.79 ± 0.13*** | 1.17 ± 0.08^###,&&&^ |
| HDL-C (mM) | 0.90 ± 0.13 | 0.56 ± 0.05* | 1.29 ± 0.13** | 0.77 ± 0.17^###,&^ |
| LDL-C (mM) | 0.49 ± 0.04 | 0.31 ± 0.05* | 0.86 ± 0.07*** | 0.49 ± 0.07^###,&&&^ |
| Tumor TG  (mg/mg protein) | 84.76 ± 18.12 | 72.59 ± 8.45 | 235.33 ± 41.86*** | 97.44±12.01^###^ |
| Tumor FFA  (mmol/g protein) | 17.20 ± 3.17 | 10.54±1.28** | 26.12 ± 4.00*** | 13.35 ± 1.13^###^ |

Blood and tumor tissues were collected from Figure 2A. Serum levels of TG, FFA, T-CHO, HDL-C, LDL-C were measured by an automated biochemical analyzer. Levels of TG and FFA in tumor tissues were determined by indicated assay kits. mean ± SEM; *p < 0.05; **p < 0.01; ***p < 0.001 vs control group; ^#^p < 0.05; ^##^p < 0.01; ^###^p < 0.001 vs HFD-NS group; ^&^p < 0.05; ^&&^p < 0.01; ^&&&^p < 0.001 vs NC-Pita group (n = 5); Pita: pitavastatin; NS: normal saline.

**Table S3 The lipids lowing effects of pitavastatin was impaired in CD36^-/-^ mice**

| Groups | Wild type mice | | CD36^-/-^ mice | |
| --- | --- | --- | --- | --- |
|  | NS | Pita | NS | Pita |
| Serum TG (mM) | 1.85 ± 0.14 | 1.26 ± 0.12*** | 1.04 ± 0.06*** | 0.85 ± 0.07^#,&&&^ |
| Serum FFA (U/L) | 2.07 ± 0.16 | 1.43 ± 0.15*** | 1.23 ± 0.26*** | 0.93 ± 0.12^#,&&&^ |
| T-CHO (mM) | 1.91 ± 0.27 | 1.43 ± 0.16** | 1.19 ± 0.14*** | 0.80 ± 0.12^#,&&&^ |
| HDL-C (mM) | 1.29 ± 0.09 | 1.11 ± 0.12* | 0.79 ± 0.08*** | 0.60 ± 0.12^#,&&&^ |
| LDL-C (mM) | 1.08 ± 0.16 | 0.81 ± 0.07** | 0.76 ± 0.09*** | 0.54 ± 0.15^#,&&&^ |
| Tumor TG  (μg/mg protein) | 289.35 ± 38.57 | 98.60 ± 16.76*** | 91.07 ± 9.14*** | 62.56 ± 16.27^#,&^ |
| Tumor FFA  (μmol/g protein) | 41.13 ± 10.72 | 25.05 ± 6.38* | 18.80 ± 7.15*** | 5.46 ± 4.15^#,&&&^ |

Blood and tumor tissues were collected from Figure 6A. Serum levels of TG, FFA, T-CHO, HDL-C and LDL-C were measured by an automated biochemical analyzer. Levels of TG and FFA in tumor tissues were determined by indicated assay kits. mean ± SEM; *p < 0.05; **p < 0.01; ***p < 0.001 vs control group; ^#^p < 0.05; ^##^p < 0.01; ^###^p < 0.001 vs NS-treated group in CD36^-/-^ mice; ^&^p < 0.05; ^&&^p < 0.01; ^&&&^p < 0.001 vs Pita- treated group in wild type mice (n = 7); Pita: pitavastatin; NS: normal saline.
