# Supplementary material for: CD36 inhibition reduces non-small-cell lung cancer development through AKT-mTOR pathway
Source: Cell Biol Toxicol. 2024 Feb 6;40(1):10. doi: 10.1007/s10565-024-09848-7 (PMC10847192; doi:10.1007/s10565-024-09848-7)

**CD36 inhibition reduces non-small cell lung cancer development through AKT-mTOR pathway**

**Original and uncropped Western blot bands in this study**

**
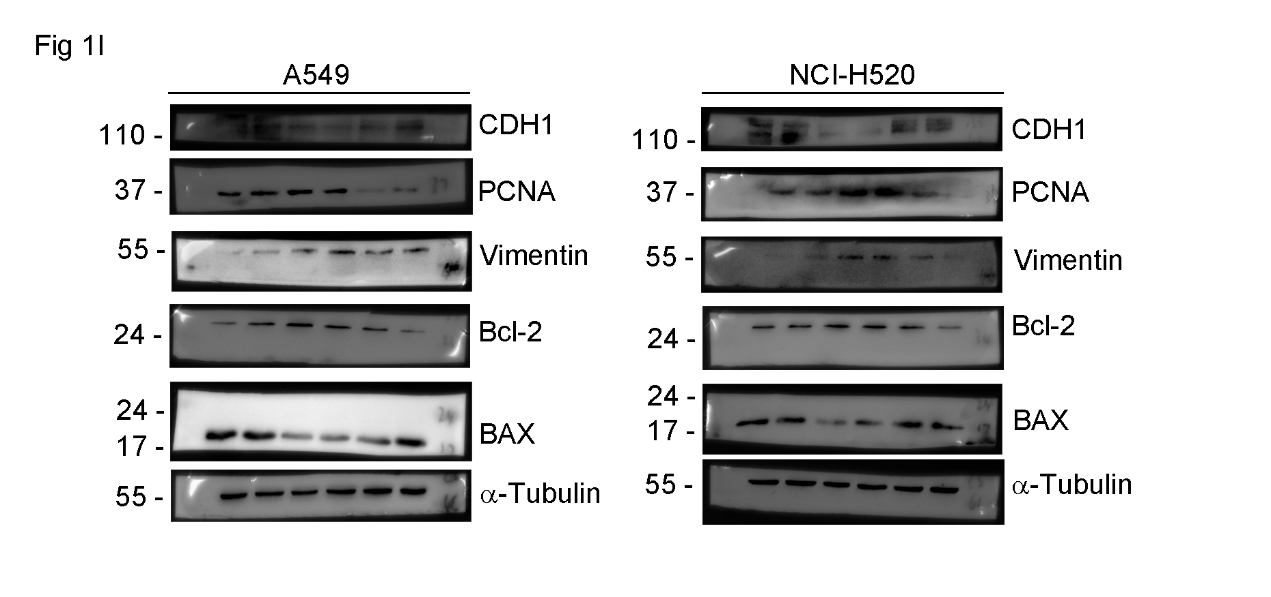
**

**
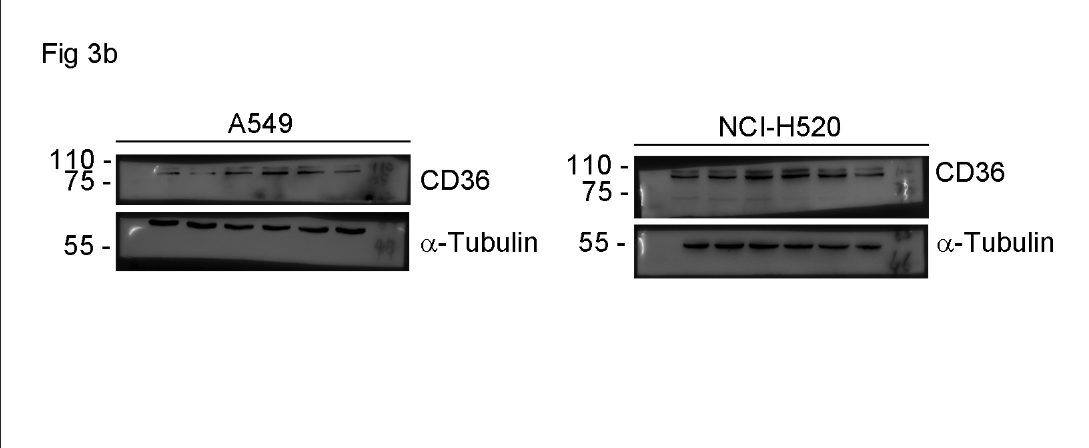
**

**
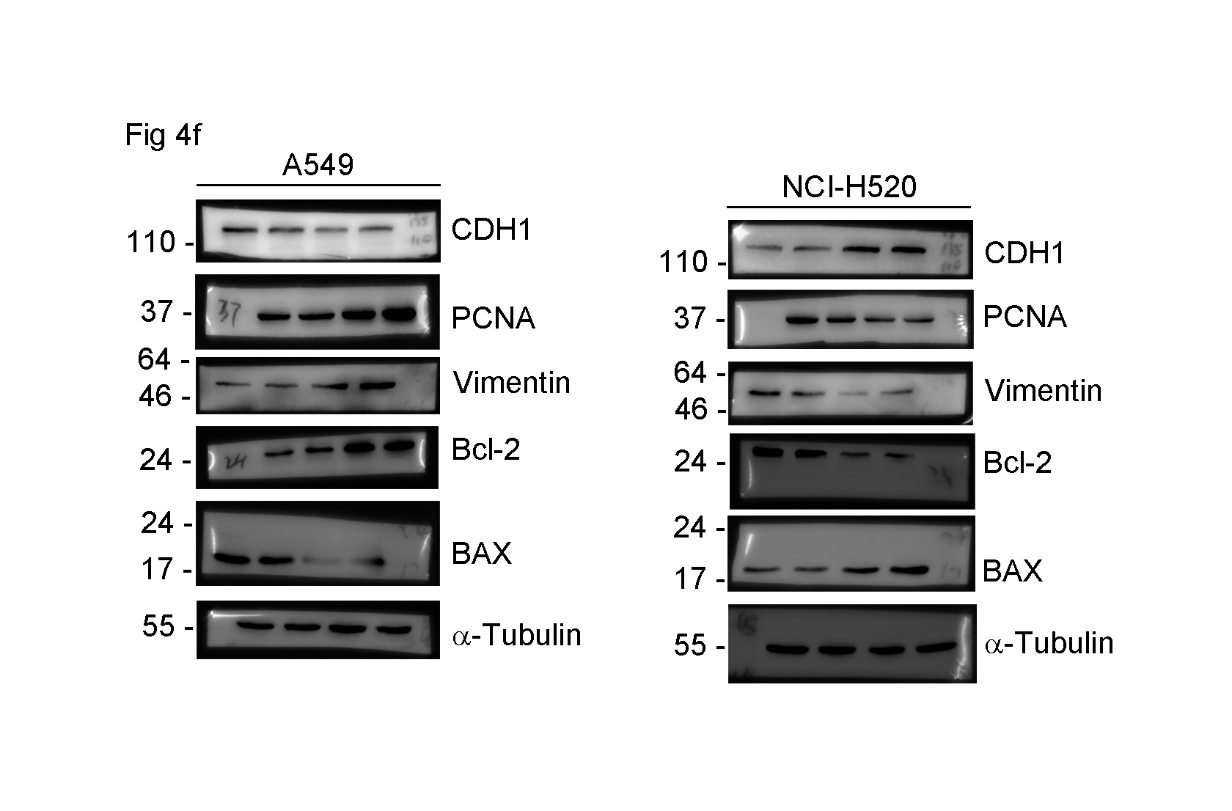
**

**
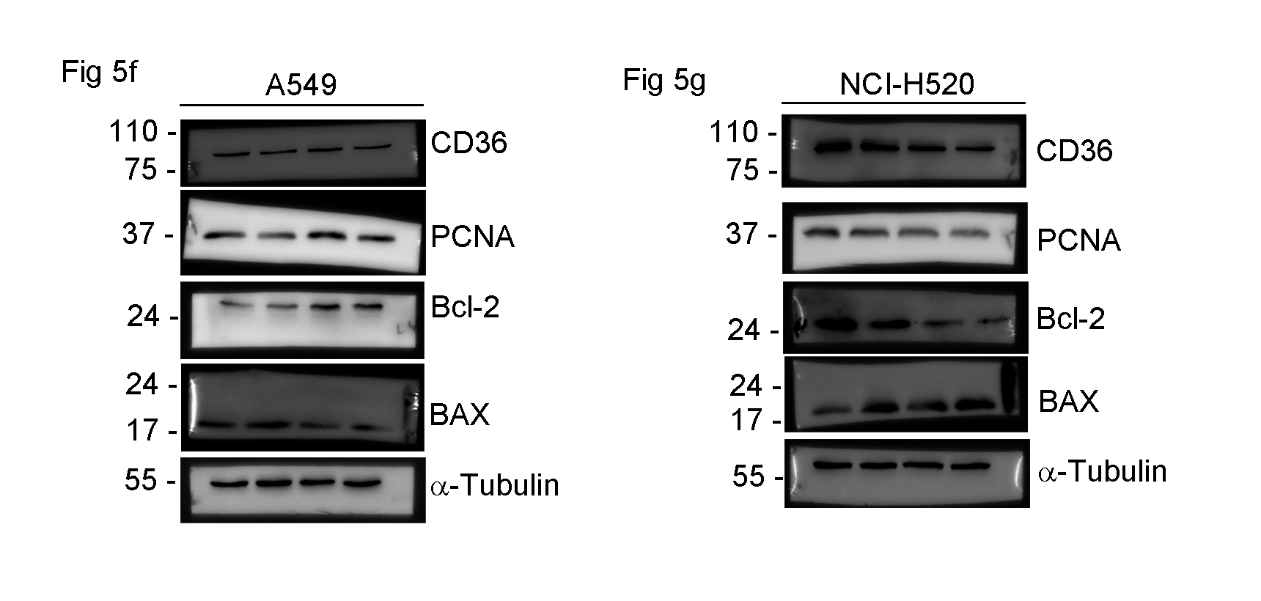
**


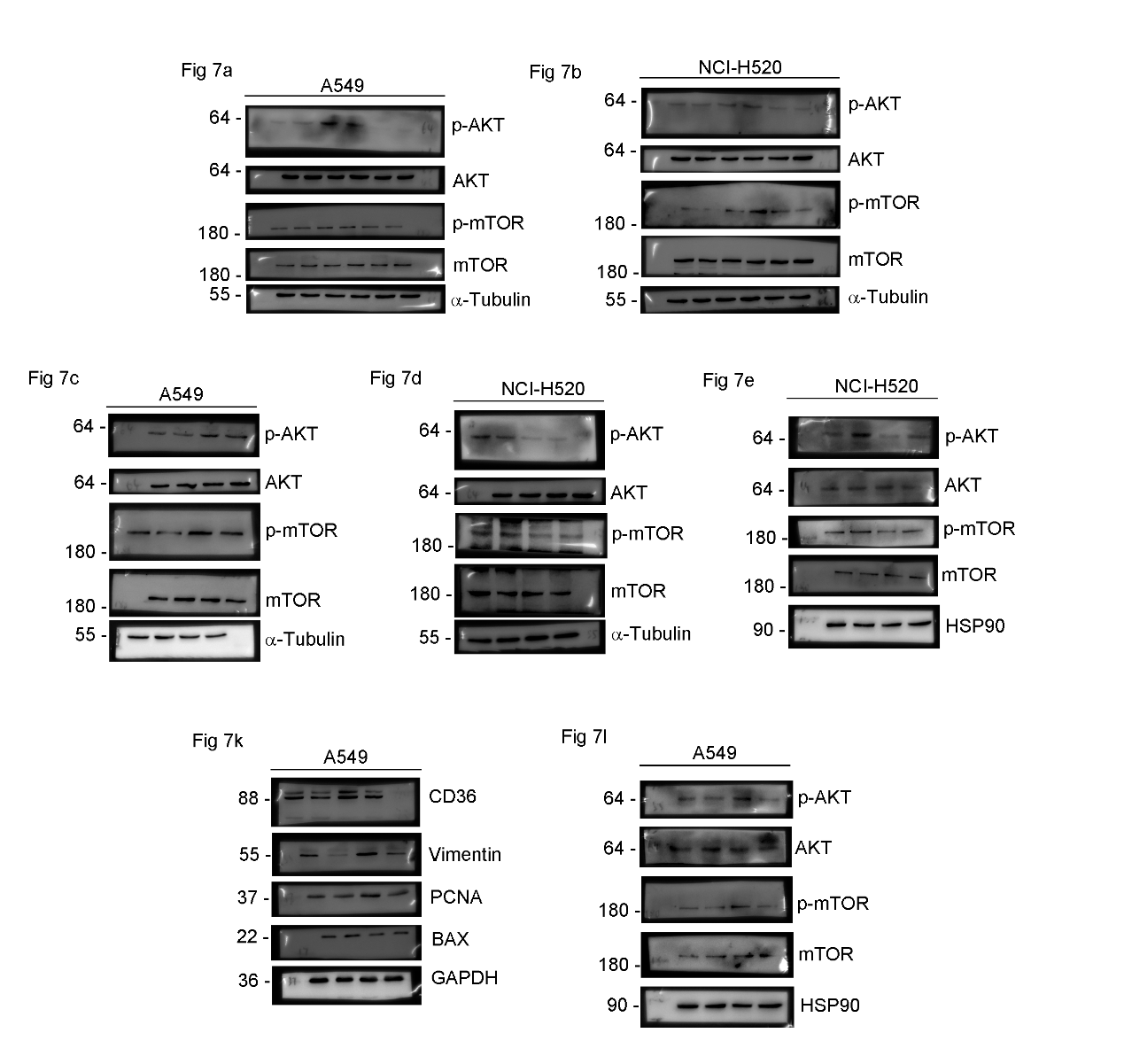


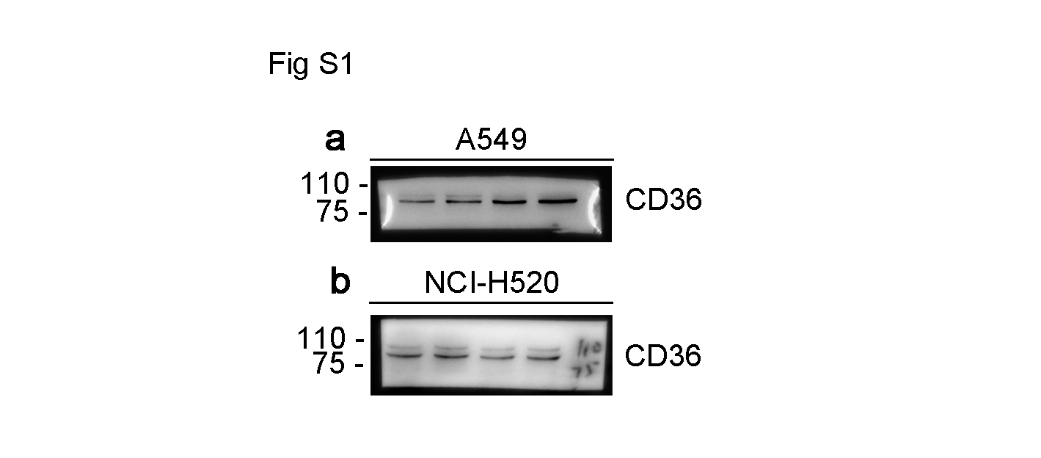


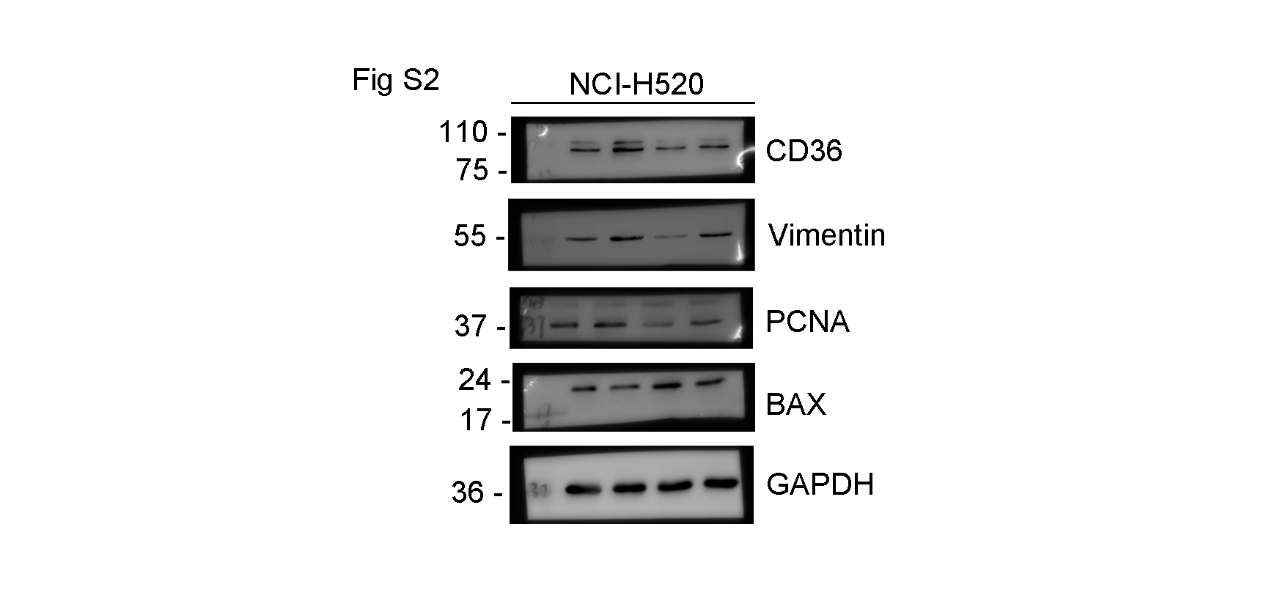

Supplement: Supplementary file 3 — Supplementary file3 (DOC 1270 KB) [file 10565_2024_9848_MOESM3_ESM.doc]
